# Ruthenium Nitrosyl Complexes with Bidentate Heterocycles and Chloride Ligands: Synthesis and Photorelease of NO

**DOI:** 10.3390/ijms27146172

**Published:** 2026-07-10

**Authors:** Anastasia O. Brovko, Ivan A. Yakovlev, Natalia V. Kuratieva, Dmitriy G. Sheven, Gennadiy A. Kostin

**Affiliations:** 1 Nikolaev Institute of Inorganic Chemistry, Siberian Branch of the Russian Academy of Sciences, 3 Acad. Lavrentiev Avenue, 630090 Novosibirsk, Russia; a.brovko@g.nsu.ru (A.O.B.); yakovlev@niic.nsc.ru (I.A.Y.); kuratieva@niic.nsc.ru (N.V.K.); dmitriy.sheven@ya.ru (D.G.S.); 2 N.N. Vorozhtsov Novosibirsk Institute of Organic Chemistry, Siberian Branch of the Russian Academy of Sciences, 9 Acad. Lavrentiev Avenue, 630090 Novosibirsk, Russia

**Keywords:** ruthenium nitrosyl, nitric oxide release, photochemistry, ruthenium complex

## Abstract

A set of new coordination compounds of the formula [RuNO(L)Cl_3_], with L representing the bidentate ligands bipyrimidine (bpym), bipyridine (bpy), or phenanthroline (phen), was prepared and fully characterized. For the isomeric species fac-[RuNO(bpym)Cl_3_] (**1**), fac-[RuNO(phen)Cl_3_] (**2**), mer-[RuNO(phen)Cl_3_] (**3**), fac-[RuNO(bpy)Cl_3_] (**4**), and mer-[RuNO(bpy)Cl_3_] (**5**), an evaluation of the photoresponse in DMSO was performed under excitation with 450 nm using a continuous-flow arrangement that enabled the simultaneous acquisition of IR and UV-Vis spectral data. Quantum yields for photoinduced release of NO were determined as follows: 5.4 ± 0.5% for fac-[RuNO(bpym)Cl_3_] (**1**); 0.8 ± 0.1% for fac-[RuNO(phen)Cl_3_] (**2**); 0.6 ± 0.1% for mer-[RuNO(phen)Cl_3_] (**3**); 3.7 ± 0.1% for fac-[RuNO(bpy)Cl_3_] (**4**); 2.1 ± 0.1% for mer-[RuNO(bpy)Cl_3_] (**5**). The product of the photolysis of compound **5** in acetonitrile solution was isolated and structurally characterized as mer-[Ru(CH_3_CN)(bpy)Cl_3_] (**6**). Taking into account the presence of typical DNA intercalating ligands, the complexes can be potentially exploited as photoNORMs.

## 1. Introduction

Cancer remains one of the leading causes of mortality worldwide, with an estimated 19.3 million new cases and almost 10 million deaths reported globally in 2020 [1]. Despite the clinical success of platinum-based chemotherapeutics, inaugurated by cisplatin [2], their use is hampered by severe systemic toxicity and intrinsic and acquired resistance, as well as a narrow spectrum of activity. This has stimulated an intensive search for alternative metal-based anticancer agents [3].

**Scheme 1:**
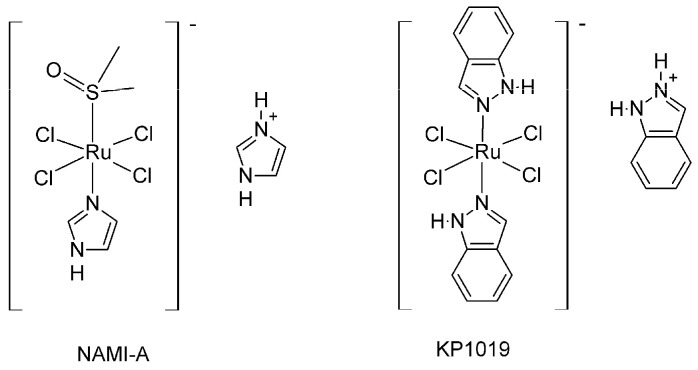
Chemical structures of NAMI-A amd KP1019.

Among non-platinum candidates, ruthenium complexes have attracted considerable interest. Their octahedral geometry, accessible Ru(II)/Ru(III)/Ru(IV) redox couples, ligand exchange kinetics comparable to those of Pt(II), and propensity for transferrin- and albumin-mediated tumour delivery make them suitable scaffolds for drug design [4,5]. Several Ru(III) complexes have advanced into human clinical trials, including imidazole complex NAMI-A {(ImH)[trans-RuCl_4_(DMSO)(Im)]} [5], indazole complex KP1019 {(IndH)[trans-RuCl_4_(Ind)_2_]} (Scheme 1), and its more soluble sodium analogue NKP 1339/BOLD 100 [6]. Moreover, the Ru(II) polypyridyl photosensitiser TLD 1433 has reached phase II clinical evaluation as a photodynamic agent against non-muscle-invasive bladder cancer [7]. These advances illustrate both the therapeutic versatility of the ruthenium platform and the growing relevance of light-activated metal complexes for delivering spatiotemporally controlled cytotoxicity.

A complementary strategy for combating malignant tissue exploits the multifaceted biology of nitric oxide (NO), a small free-radical messenger whose role in vasodilation, neurotransmission and host defence was recognized by the 1998 Nobel Prize in Physiology or Medicine [8]. The action of NO is strongly concentration-dependent [9,10,11] and NO also has a very short half-life in tissues (seconds) and indiscriminate reactivity, which together call for prodrugs that liberate NO only at the desired site and time. There is a large variety of compounds capable of forming NO-releasing molecules (NORM), including different organic compounds (furoxans, nitrites, nitrobenzenes, etc.) and metal nitrosyls [12].

Photoactivated nitric oxide-releasing molecules (photoNORMs) address this requirement by coupling NO delivery to an optical trigger, allowing the dose to be tuned in real time and confined to the irradiated volume [13]. Within this family, ruthenium nitrosyl complexes are particularly attractive. Most {RuNO}^6^ species are diamagnetic and kinetically inert in the dark, yet undergo clean photodissociation of NO^•^ with the concomitant formation of a solvento-Ru(III) fragment [14,15]. The wavelength, quantum yield and chemoselectivity of the Ru–NO photolysis are extremely sensitive to the ancillary ligand sphere.

Halide co-ligands, and chloride in particular, play a dual role in this design: they stabilise the {RuNO}^6^ core through strong σ- and π-donation, modulate the energy of the NO-dissociative metal-to-ligand/ligand-to-metal charge transfer (MLCT/LMCT) manifold, and give rise, after photolysis, to chlorido-/aqua-Ru(III) species that may themselves act as DNA-binding cytotoxins, in close analogy to the activation pathway of NAMI-A and KP1019 [5,16].

Ruthenium nitrosyl complexes bearing chloride and various N-donor ligands, including bipyridine and phenanthroline, were first synthesized and characterized as early as 1966 by Fairy and co-workers, starting from RuNOCl_3_ [17]. Subsequently, the structures of two geometric isomers of [RuNO(bpy)Cl_3_] were determined and described by Haukka et al. in 1995 [18]. Nevertheless, the photoinduced release of NO from these complexes has not been investigated.

Building on these considerations, we report the synthesis and comprehensive characterization of a new family of charge-neutral ruthenium nitrosyl complexes in which the inner coordination sphere contains chloride ligands together with phenanthroline (phen), bipyridine (bpy) or bipyrimidine (bpym). The photochemical behavior under monochromatic 450 nm LED irradiation was studied using a flow-through cuvette system that permits the simultaneous acquisition of IR and UV spectral series. ESI mass spectrometry was employed to identify the resulting ruthenium-containing photoproducts. Altogether, the collected data provide a coherent picture of how the chloride ligand environment governs the photochemistry and physicochemical profile of {RuNO}^6^ photoNORMs and, in our view, offer useful design guidelines for the next generation of light-activated anticancer agents that deliver nitric oxide.

## 2. Results and Discussion

### 2.1. Synthesis and Crystal Structures

The new complexes **1**–**3** and the earlier reported complexes **4**–**5** were prepared using a similar technique. A standard pathway to the synthesis of novel ruthenium nitrosyl compounds was based on K_2_[RuNOCl_5_] as the starting reagent, prepared from ruthenium(III) chloride [19]. The synthesis of trans-[RuNO(tpy)Cl_2_]PF_6_ reported by Hirano and colleagues [20] served as the starting point for the preparation of compounds with polydentate ligands. However, in contrast to the reported synthetic procedure for terpyridine, the addition of potassium chloride had no effect on the reaction yield in the case of the polydentate ligands presented in this work.

Upon the addition of 1 equivalent of bidentate ligand (bpym/phen/bpy) to K_2_[Ru(NO)Cl_5_], the complexes [RuNO(L)Cl_3_] are formed. It should be noted that in the case of bipyrimidine and bipyridine, the fac-isomer (fac-[RuNO(bpym)Cl_3_] (**1**) and fac-[RuNO(bpy)Cl_3_] (**4**)) are obtained first, whereas phenanthroline affords the mer-isomer (mer-[RuNO(phen)Cl_3_] (**3**)). The formation of different geometrical isomers in the solid state has been reported previously by our research group. Specifically, bipyrimidine and bipyridine yield one complex isomer, while phenanthroline yields the other in the reaction with [RuNO(NH_3_)_2_(NO_3_)_3_] [21].

**Figure 1:**
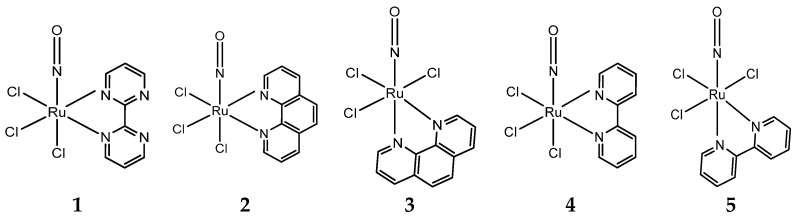
Compound structures in the present study.

Interestingly, other geometrical isomers for phenanthroline and bipyridine were also isolated. For instance, fac-[RuNO(phen)Cl_3_] (**2**) was obtained from the mother liquor after filtration of compound **3**, while the mer-isomer of the bipyridine analogue, mer-[RuNO(bpy)Cl_3_] (**5**), was formed upon the addition of two equivalents of bpy to K_2_[Ru(NO)Cl_5_]. The structures of compounds **1**–**5** are given in Figure 1.

### 2.2. Structural Information for Complexes ***1***–***3***

The structures of fac-[RuNO(bpym)Cl_3_] (**1**), fac-[RuNO(phen)Cl_3_] (**2**), mer-[RuNO(phen)Cl_3_] (**3**) were determined by single-crystal XRD. The crystal structures of bipyridine compounds **4** and **5** were reported earlier by Haukka et al. [18].

The principal interatomic distances determined for complexes **1**–**3** are compiled in Table 1 and prove to be fully consistent with the values generally observed for this family of compounds. Notably, the Ru–N–O moiety adopts a near-linear geometry, with the Ru–NO and N–O bond lengths falling in the ranges of 1.74–1.75 and 1.13–1.14 Å, respectively. The Ru–N(L) bond lengths, which are around 2.1 Å, are characteristic of ruthenium centers coordinated by nitrogen-donor ligands.

All compounds whose crystal structures are reported herein display similar packing arrangements. The presence of the nitrosyl group, the organic aromatic H-donor ligand, and the chloro ligands coordinated with the ruthenium means that Cl···H and O···H interactions were the dominant forces in the crystal packing. Hirshfeld surface and fingerprint plots were calculated (Figures S1–S3). The prevalence of hydrogen-inclusive intermolecular contacts is evidenced by the characteristic spikes in the fingerprint plots: 23.9% Cl-H, 11.0% O-H and 11.0% N-H interactions in **1**; 27.1% Cl-H, 16.6% C-H and 9.4% O-H interactions in **2**; 42.0% Cl-H, 10.4% C-H and 9.2% O-H interactions in **3**.

**Figure 2:**
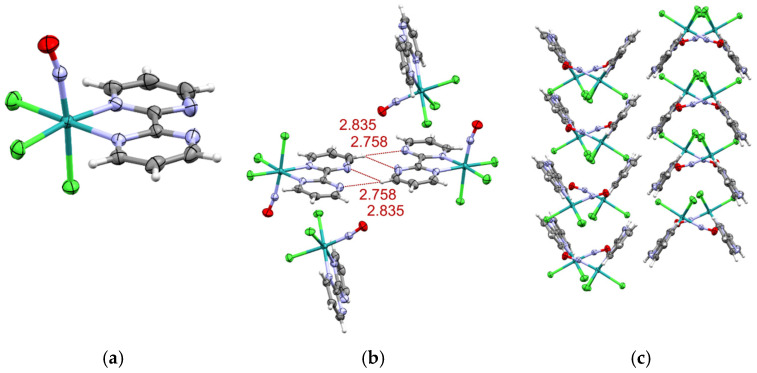
Structural fragment of fac-[RuNO(bpym)Cl_3_] (**1**) (panel (**a**)), crystallographic unit cell of **1** (panel (**b**)) and crystal packing of **1** (panel (**c**)). ADPs are given at the 50% probability level.

In the crystal structure of compound **1**, molecules are paired through C–H···N interactions between two adjacent bipyrimidine ligands, with N···H distances of 2.758 Å and 2.835 Å (Figure 2b). The bipyrimidine moieties of each pair are coplanar. These pairs are arranged into layers with an interlayer spacing of 3.080 Å and a lateral offset, characteristic of slipped π–π stacking (the centroid-to-centroid distance is 3.713 Å). Adjacent molecules, as depicted in Figure 2b, are oriented at an angle of 70.77°, forming identical pairs with the same N···H distances and interlayer separations. This arrangement gives rise to a bilayer packing motif of inverted layers in the crystal structure (Figure 2c).

**Figure 3:**
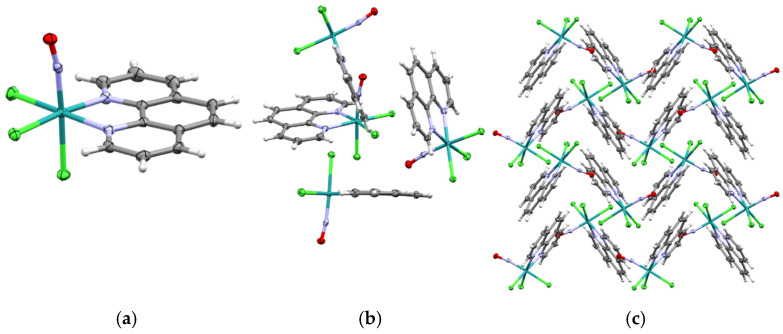
Structural fragment of fac-[RuNO(phen)Cl_3_] (**2**) (panel (**a**)), crystallographic unit cell of **2** (panel (**b**)) and crystal packing of **2** (panel (**c**)). ADPs are given at the 50% probability level.

In the unit cell of compound **2**, the phenanthroline ligand planes of all four molecules are non-parallel, with angles of 23.01°, 66.80°, and 71.68°. This arrangement gives rise to a herringbone-like packing motif parallel to the ab plane (Figure 3c).

**Figure 4:**
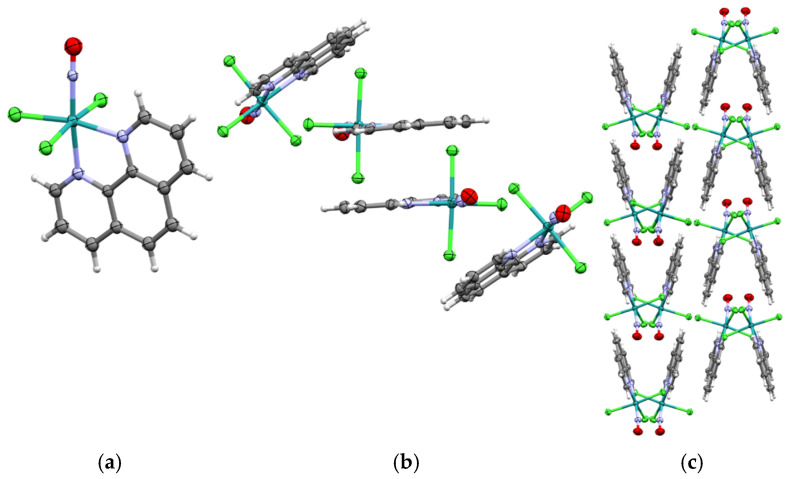
Structural fragment of mer-[RuNO(phen)Cl_3_] (**3**) (panel (**a**)), crystallographic unit cell of **3** (panel (**b**)) and crystal packing of **3** (panel (**c**)). ADPs are given at the 50% probability level.

In the unit cell of compound **3** (Figure 4), pairs of molecules with parallel phenanthroline ligands form parallel planes with π–π stacking at 3.454 Å. The adjacent phenanthroline plane is rotated by an angle of 31.55° relative to the previous phenanthroline plane. As a result, a bilayer packing motif of the crystal structure is formed, parallel to the ab plane.

### 2.3. Stability in DMSO Solutions

Since further photochemical studies were carried out in DMSO solutions, it should be noted that the geometry of all crystalline products is preserved upon dissolution. For NMR spectroscopic investigations, samples were prepared by dissolving crystalline products **1**–**5** in DMSO d_6_. The corresponding ^1^H NMR spectra are provided in the Supplementary Information (Figures S4–S8). The ^1^H NMR spectra of all investigated complexes exhibit characteristic signals in the range of 8–10 ppm, corresponding to the aromatic protons of the ligands.

In all fac-isomers (**1**, **2**, **4**), a mirror plane can be identified, which passes perpendicular to the plane of the aromatic organic ligand. Consequently, as in the case of the free ligand, signals corresponding to pairwise equivalent proton groups are observed with the downfield shift upon coordination with the metal center (Figures S4, S5 and S7). In contrast, for the mer-isomers, i.e., compounds **3** and **5**, the number of signals in the ^1^H NMR spectrum corresponds to the number of protons in the organic ligand, since all protons are inequivalent (Figures S6 and S8). In other words, in the mer-isomers, one coordination site of the bidentate ligand is in the cis-position to the nitrosyl group, while the other is in the trans-position, which results in inequivalence of the protons. In the fac-isomers, however, both coordination sites of the bidentate ligand are in cis-position to the nitrosyl group.

### 2.4. Photoinduced NO Release

The experiments were carried out on solutions with a concentration of 10^−3^ M in DMSO to track the UV-Vis and IR spectral changes accompanying NO release. For all five complexes, irradiation leads to a decrease in the intensity of the ν(NO) band in the IR spectra and an increase in absorption in the 300–500 nm region of the UV-Vis range—features characteristic of photo-processes involving nitric oxide release [13,14,22]. In the dark conditions, the photochemical NO cleavage is absent, as confirmed by the constancy of the NMR and electronic spectra.

**Figure 5:**
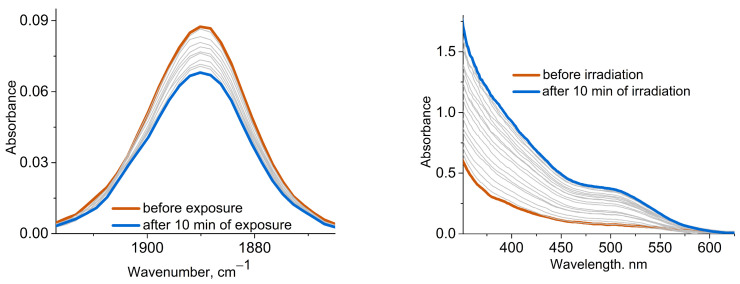
Time-dependent IR (**left**) and UV-Vis (**right**) spectra for DMSO solution of complex fac-[RuNO(bpym)Cl_3_] (**1**) under continuous 450 nm excitation for 10 min.

As the decrease in the characteristic IR band is a direct consequence of Ru–NO bond-breaking and the accompanying reduction in the reagent concentration, the normalized areas of this vibrational band can be treated as proportional to the nitrosyl complex concentration at the time of irradiation. This method allows the reagent concentration to be derived directly from the IR spectral data. Based on the time-dependent reagent concentration, the corresponding contribution to the UV-Vis absorbance can be accounted for. The experimental data set for compound **1** is presented in Figure 5, while the experimental data sets for compounds **2**–**5** are provided in the Supplementary Materials (Figures S9–S12).

The quantum yields of photodissociation for compounds **1**–**5** were determined from the first 20 data points (recorded at 30 s intervals) and are listed in Table 2.

The measured quantum yields after 450 nm excitation fall within the typical interval reported for ruthenium nitrosyl species bearing N-heterocyclic donor ligands [22,23]. For complex **5**, photolysis was also performed in acetonitrile instead of DMSO. Upon solvent replacement, no significant changes are observed in either the IR or optical spectra, but the quantum yield changes slightly, which is attributed to the higher acceptor number of acetonitrile [24,25].

### 2.5. TD-DFT Calculations

A trend is observed toward higher quantum yield values for the facial isomers of compounds **2**–**5** compared to the meridional ones—they were slight for the phenanthroline complex and nearly twofold for the bipyridine compound. TD-DFT calculations were performed to analyze the electronic transitions and molecular orbitals of these compounds. The general view of UV-VIS spectra was analyzed and the transitions with calculated wavelengths in the neighborhood of 450 ± 50 nm were selected. Since the main contribution to the NO release is related to the transitions to Ru-NO antibonding orbitals, these transitions were further taken into consideration among those selected. In all considered complexes, LUMO+1, +2 are located on the Ru-NO coordinate and have an antibonding Ru-NO character, while LUMO is mostly located on chloride ligands and LUMO+3 is predominantly located on organic ligands.

Among the investigated complexes, compound **1** exhibited the highest quantum yield. The orbital contributions are summarized in Table S1 (Supplementary Materials). The calculated transitions closest to the irradiation wavelength occur at 441.0, 436.9, 430.2, 427.3, and 426.2 nm. The enhanced quantum yield of complex **1** may be partly attributed to the better energetic match between the excitation wavelength and the excited states that possess a Ru–NO antibonding character, thereby facilitating the more efficient population of NO-dissociative states.

**Figure 6:**
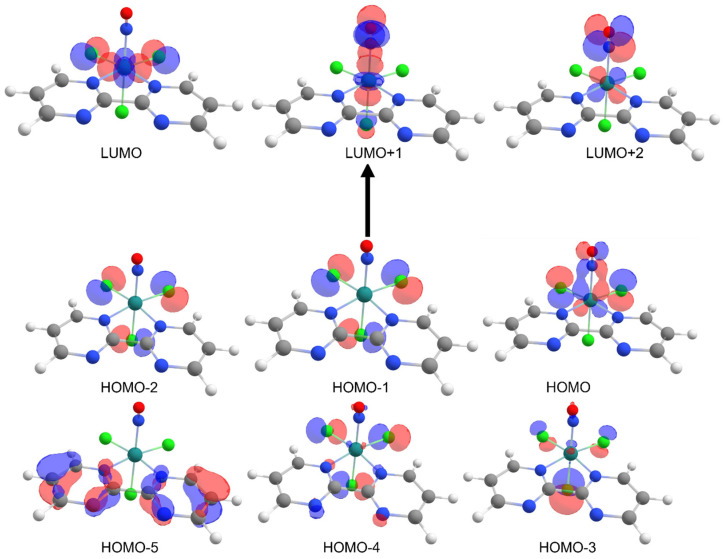
Main orbitals contributing to the transitions for fac-[RuNO(bpym)Cl_3_] (**1**).

Molecular orbital analysis indicates that the relevant excitations involve electron density transfer, primarily from chloride-centered orbitals (HOMO–1 to HOMO–4) or from bipyrimidine ligand (HOMO–5) (Figure 6). The HOMO–1 orbital is delocalized over the chloride ligands, the ruthenium center, and the nitrosyl ligand. For complex **1**, the LUMO orbital is located on the ruthenium atom and two chloride ligands, while the LUMO+1 and LUMO+2 possess substantial electron density along the Ru–NO bond coordinate and are likely the most relevant acceptor orbitals for the NO photo release pathway owing to their pronounced Ru–NO antibonding character. The transitions to all the described orbitals exhibit a mixed LMCT/MLCT character. Accordingly, the transitions at 436.9, 430.2, and 426.2 nm are expected to contribute most significantly to NO photo release, as they populate LUMO orbitals possessing substantial Ru–NO antibonding character. Owing to the significant broadening of the absorption bands, transitions located close to the irradiation wavelength may influence the photolysis efficiency not only by increasing the fraction of absorbed photons, but also by increasing the probability of populating excited states with Ru–NO antibonding character. In contrast, more intense transitions located at shorter wavelengths contribute less under 450 nm irradiation because of the lower efficiency of the excitation. The transition at 423.7 nm to the LUMO+3 orbital transfers electron density predominantly to π* orbitals localized on the bipyrimidine ligand and is therefore expected to contribute less efficiently to NO photo release.

Comparison of the two isomers **2** and **3**, which contain the phenanthroline ligand reveals that both compounds exhibited relatively low quantum yields, with a slight advantage for the facial isomer. The main transitions involved in the excitation are given in Table S2 (Supplementary Materials).

**Figure 7:**
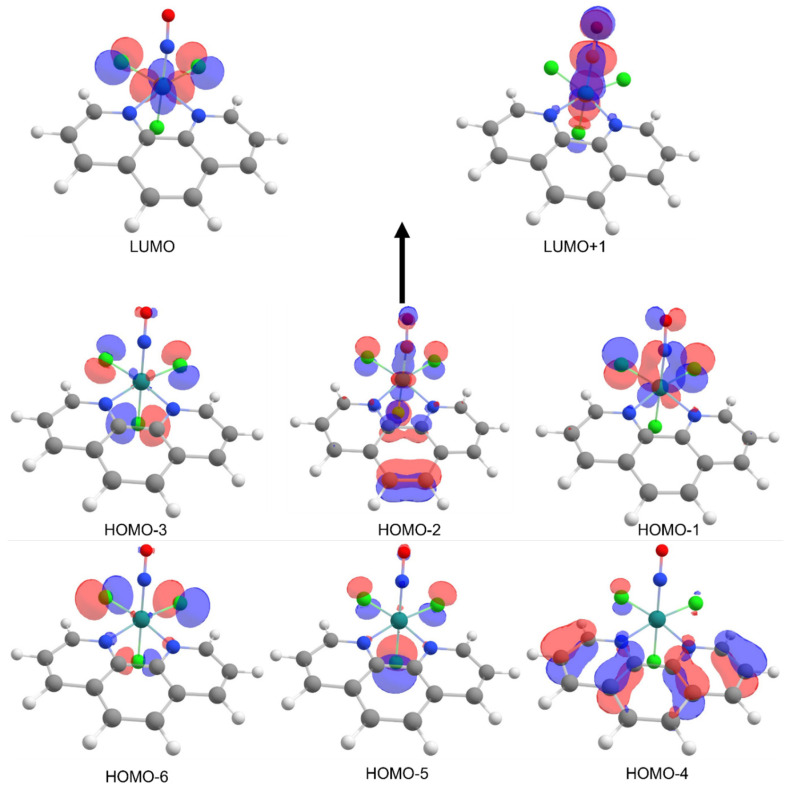
Main orbitals contributing to the transitions for fac-[RuNO(phen)Cl_3_] (**2**).

**Figure 8:**
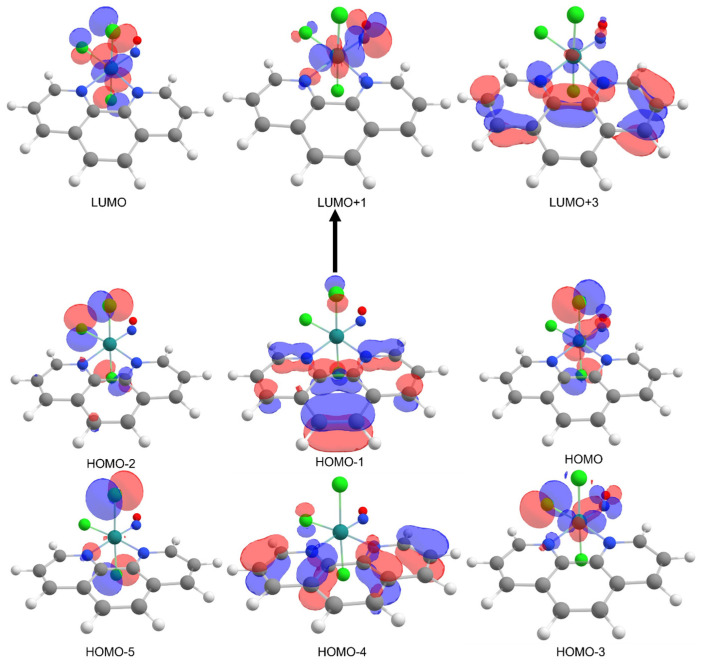
Main orbitals contributing to the transitions for mer-[RuNO(phen)Cl_3_] (**3**).

The HOMO–x orbitals are involved in the transitions for both isomers (Figure 7 and Figure 8). For the fac-complex, these are predominantly orbitals located on the chloride ligands, except for the HOMO–1 (Cl^−^, Ru, and nitrosyl group) and HOMO–5 (phenanthroline) orbitals. For the mer-isomer, these contributions come from orbitals situated on phenanthroline (HOMO–2, HOMO–5) and on the Ru–NO unit (HOMO–1, HOMO–4). The transitions for the fac-isomer are to an antibonding LUMO orbital, which is located on the Ru atom and two chloride ligands, and to an antibonding LUMO+1 orbital that exhibits the classic localization along the Ru–NO coordinate. Consequently, transitions involving this orbital are expected to be the most relevant to the photodissociation pathway. For the mer-isomer, transitions in this region are possible to a LUMO orbital that is structurally similar to that of the fac-isomer, and to the LUMO+1 and LUMO+3 orbitals. Notably, transition to LUMO+1 populates an excited state possessing significant Ru–NO antibonding character and is therefore expected to facilitate NO photorelease, since LUMO+3 is localized on the phenanthroline orbitals.

Analysis of the transitions shows that, for the mer-isomer, only the transitions at 457, 408, and 407.5 nm may play an important role in photolysis, and for the fac-isomer, those at 520, 424.9, and 408.6 nm are important—i.e., transitions to the LUMO+1 orbital. It is also essential to take into account the relative oscillator strength of the transitions that contain the orbitals required for photodissociation. As a qualitative descriptor, we estimated the relative contribution of transitions populating Ru–NO antibonding orbitals by weighting their orbital contribution with the corresponding oscillator strength RC = Σa_i_f_i_/Σf_i_, where f_i_ is the oscillator strength and a_i_ is the share of transitions resulting in an antibonding NO population in the range 500–400 nm. Although this descriptor has no rigorous mechanistic basis, it qualitatively follows the observed trend in quantum yields for fac-mer isomers in compounds **2**–**5** (see Table 3).

The overall low quantum yield for phenanthroline complexes may be associated with the presence of numerous ligand-centered excited states involving phenanthroline orbitals, which can provide additional non-dissociative relaxation pathways competing with NO release [26].

**Figure 9:**
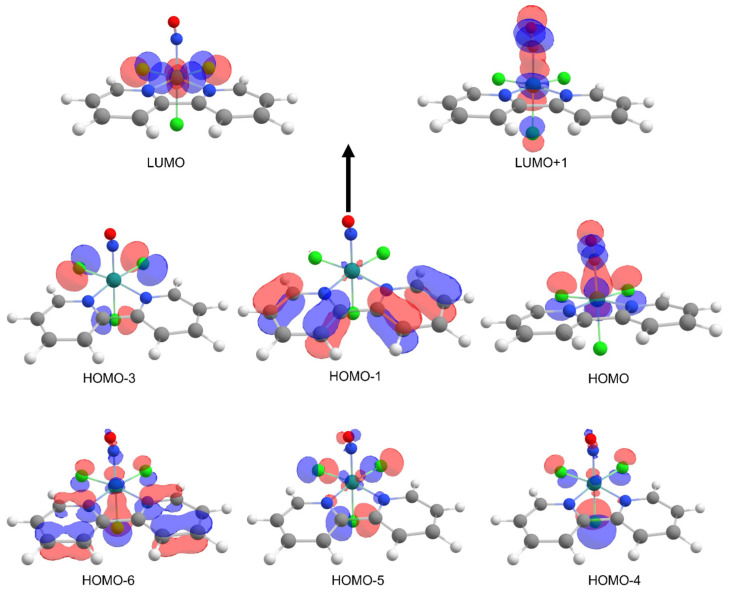
Main orbitals contributing to the transitions for fac-[RuNO(bpy)Cl_3_] (**4**).

**Figure 10:**
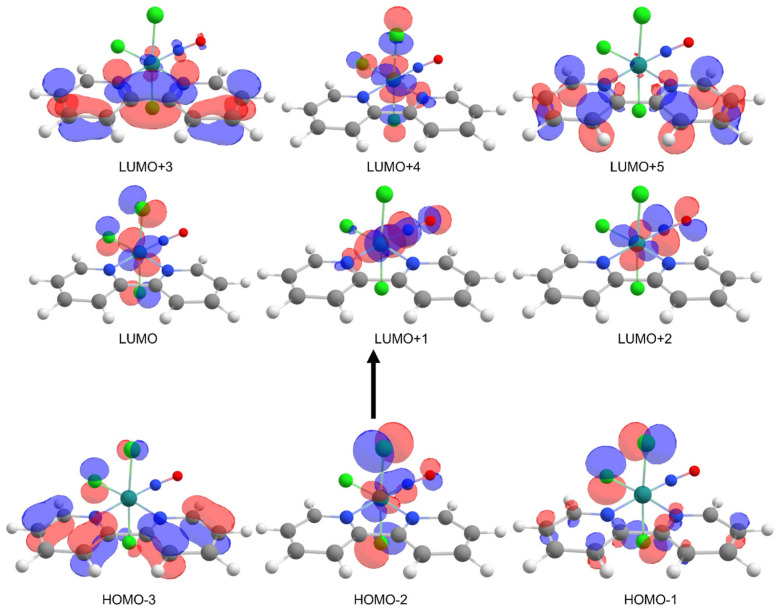
Main orbitals contributing to the transitions for mer-[RuNO(bpy)Cl_3_] (**5**).

A similar analysis was performed for compounds **4** and **5**, the fac- and mer-isomers containing bipyridine (Figure 9 and Figure 10). The main transitions involved in the excitation are given in Table S3 (Supplementary Materials).

For the fac-isomer, the transition pattern is similar to that previously discussed for bpym. The transitions are fairly distant from the irradiation wavelength; however, there is a large contribution from transitions to the required antibonding LUMO+1 orbital, located along the Ru–NO bond coordinate. For the mer-isomer, the picture is somewhat different: the transitions are much closer to 450 nm, but a very large contribution comes from LLCT transitions to chloride ligands and pyridine rings, which do not lead to photodissociation. If one calculates the relative contribution of the required transitions to the total absorption in this region, a correlation can also be noted between this contribution and the quantum yield (see Table 3).

Overall, all compounds exhibit a fairly similar pattern of transitions in this region, differing in transition intensity and position, and are very similar in terms of their molecular orbital structure. The occupied orbitals involved in the visible-light excitations are primarily chloride-, ligand-, and Ru-centered, whereas the relevant acceptor orbitals possess significant Ru–NO antibonding character in LUMO+1, LUMO+2. Consequently, NO photorelease is expected to proceed through the population of excited states that weaken the Ru–NO bond. Variations in quantum yield therefore appear to mainly originate from differences in the energetic position and relative intensity of these excited states rather than from fundamentally different photochemical mechanisms. It should be emphasized that TD-DFT calculations describe only the initial vertical excitation process. The experimentally observed quantum yields are determined by the entire excited-state relaxation landscape, including internal conversion, intersystem crossing, vibrational relaxation and solvent-assisted processes. Therefore, the present analysis should be regarded as a qualitative description of the electronic factors governing NO photorelease.

### 2.6. Electrospray Mass-Spectroscopy of the Solutions After Photolysis

**Figure 11:**
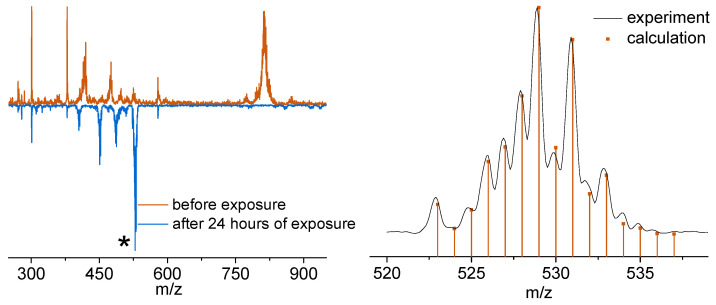
ESI-MS spectra before and after light irradiation by 450 nm of fac-[RuNO(bpym)Cl_3_] (**1**) (**left**) and the peak 529 *m*/*z* from mass spectra of **1** with calculated isotope pattern (**right**). *—the predominant form after photolysis.

**Figure 12:**
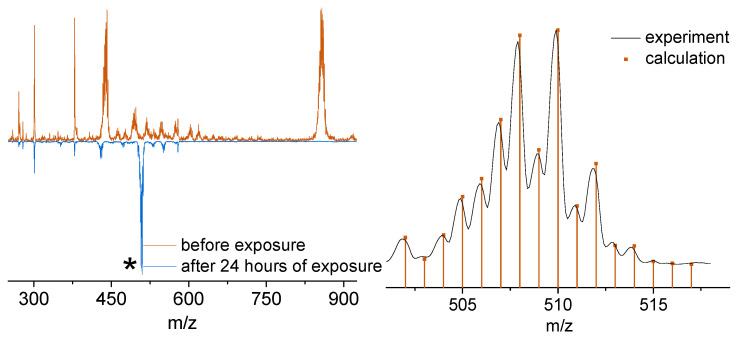
ESI-MS spectra before and after light irradiation by 450 nm of fac-[RuNO(phen)Cl_3_] (**2**) (**left**) and the peak 510 *m*/*z* from mass spectra of **2** with calculated isotope pattern (**right**). *—the predominant form after photolysis.

To gain insight into the complex species generated both directly upon irradiation and through subsequent transformations, the irradiated solutions were subjected to supplementary ESI-MS analysis. For all compounds, mass spectra with a single predominant species were obtained after 24 h of irradiation (Figure 11, Figure 12 and Figures S13–S15). These predominant species are marked with an asterisk (*), and their tentative compositions are listed in Table 4. All masses presented in the table correspond to the most abundant ruthenium isotope, ^102^Ru. The peak profile (isotope pattern) confirms the presence of ruthenium and the number of chloro-ligands in the species (Figure 11 right, Figure 12 right). The primary photolysis products for complexes **2**–**5** in DMSO solution after NO release followed by the coordination of a solvent molecule should be the neutral [Ru(L)(DMSO)Cl_3_] species. Since mass spectrometry detects charged species, it can be assumed that upon source fragmentation, one chloride ligand is replaced by a DMSO molecule, yielding the observed charged species rather than the neutral photolysis product. In case of the complex 1, the additional nitrogen atoms outside the coordination sphere can influence the formation of solvent pairs with DMSO. This could be the reason that the most abundant ion detected after the photolysis of **1** is [Ru(bpym)(DMSO)_3_Cl]^+^. Thus, all compounds reported herein are consistent with the literature, according to which NO release from ruthenium nitrosyl complexes is accompanied by the coordination of a solvent molecule at the vacated coordination site [27,28].

### 2.7. The Crystal Structure of NO Release Product for Bipyridine Complex ***5***

As mentioned above, the photochemical release of NO from compound **5** was examined in both DMSO and acetonitrile solutions. Upon irradiation of the acetonitrile solution for 24 h, single crystals of the photolysis product mer-[Ru(CH_3_CN)(bpy)Cl_3_] were obtained by slow evaporation of the solvent. The product yield is quantitative, aside from minor mechanical losses due to the small scale of the reaction.

Compound **6** shows agreement with the mechanism of Ru-NO photolysis reported in the literature [22,23,24], according to which the photoinduced liberation of nitric oxide is accompanied by the coordination of a solvent molecule at the vacant coordination site previously occupied by the nitrosyl ligand. The authors consider it most important to note that the geometry of the photolysis product corresponds to that of the nitrosyl-containing compound, namely compound **5**. The authors consider it noteworthy that the photolysis product retains the geometry of the parent nitrosyl complex (compound **5**). These data can serve as a basis for further considerations of intrinsic mechanism of photolysis and following substitution.

**Figure 13:**
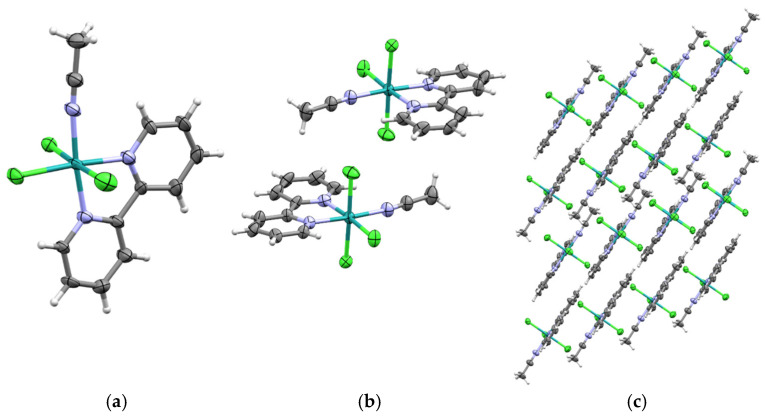
Structural fragment of mer-[Ru(CH_3_CN)(bpy)Cl_3_] (**6**) (panel (**a**)), crystallographic unit cell of 6 (panel (**b**)) and crystal packing of **6** (panel (**c**)). ADPs are given at the 50% probability level.

The most important changes in ruthenium coordination sphere after NO–acetonitrile substitution (Table 5) are related to Ru-N(bpy) distances, which shorten for 0.02–0.07 Å due to the stronger binding with NO relative to CH_3_CN. This is also confirmed by the expected increase in Ru-N(NO, CH_3_CN) distance after NO is substituted by acetonitrile for 0.12 Å. In contrast, bond lengths Ru-Cl are less influenced, and average Ru-Cl distance remains the same at the limits of these discrepancies. In the crystal packing of compound **6**, the bipyridine ligands are arranged parallel to each other with an interplanar separation of 4.103 Å and adopt a head-to-tail packing motif, wherein the acetonitrile ligand resides above one of the bipyridine rings (centroid–methyl carbon distance: 4.326 Å). These dimeric pairs form layers with a lateral shift of 0.789 Å, parallel to the (112) plane (Figure 13c).

## 3. Materials and Methods

### 3.1. Synthesis of Compounds ***1***–***5***

All reagents used were of standard-grade purity (Merck, Darmstadt, Germany) and used without additional purification. The starting ruthenium compound K_2_[RuNOCl_5_] was prepared from commercial ruthenium chloride, as described previously [19].

#### 3.1.1. Synthesis of fac-[RuNO(bpym)Cl_3_] (**1**)

The K_2_[RuNOCl_5_] 386 mg (1 mmol) sample and the ligand sample (1 mmol of bpym, 158 mg) were dissolved in a mixture of 45 mL EtOH and 15 mL H_2_O. The mixture was heated under a watch glass for 1 h. Further evaporation to 5 mL resulted in the peach precipitate of fac-[RuNO(bpym)Cl_3_] (**1**), which was filtered and dried in air. Yield was 81%.

Elemental analysis data (%) calc: C—24.3, H—1.5, N—17.7; found: C—24.4, H—1.5, N—17.4.

^1^H NMR (500 MHz, DMSO d_6_): δ 9.59 (dd, J = 5.7, 2.1 Hz, 2H, bpym), 9.50 (dd, J = 4.8, 2.1 Hz, 2H, bpym), 8.17 (dd, J = 5.7, 4.8 Hz, 2H, bpym).

IR spectroscopy: ν(NO): 1904, 1884 (KBr).

#### 3.1.2. Synthesis of mer-[RuNO(phen)Cl_3_] (**3**)

The K_2_[RuNOCl_5_] 386 mg (1 mmol) sample and the ligand sample (1 mmol of phen, 180 mg) were dissolved in a mixture of 45 mL EtOH and 15 mL H_2_O. The mixture was heated under a watch glass for 1 h. Further evaporation to 5 mL resulted in the green precipitate of mer-[RuNO(phen)Cl_3_] (**3**), which was filtered and dried in air. Yield was 63%.

Elemental analysis data (%) calc: C—34.5, H—1.9, N—10.1; found: C—35.0, H—2.1, N—10.0.

^1^H NMR (500 MHz, DMSO d_6_): δ 9.82 (dd, J = 5.3, 0.9 Hz, 1H, phen), 9.53 (d, J = 5.3 Hz, 1H, phen), 9.16 (dd, J = 8.2, 1.0 Hz, 1H, phen), 9.06 (dd, J = 8.2, 0.9 Hz, 1H, phen), 8.47 (t, J = 8.9 Hz, 1H, phen), 8.45 (t, J = 8.9 Hz, 1H, phen), 8.35 (dd, J = 8.2, 5.3 Hz, 1H, phen), 8.22 (dd, J = 8.2, 5.3 Hz, 1H, phen).

IR spectroscopy: ν(NO): 1877 (KBr).

#### 3.1.3. Synthesis of fac-[RuNO(phen)Cl_3_] (**2**)

After filtration of mer-[RuNO(phen)Cl_3_] (**3**), the liquid was allowed to evaporate at room temperature, yielding pink crystals and an orange powder, which were both identified as the same compound, fac-[RuNO(phen)Cl_3_] (**2**). Yield was 11%.

Elemental analysis data (%) calc: C—34.5, H—1.9, N—10.1; found: C—34.1, H—1.7, N—10.2.

^1^H NMR (500 MHz, DMSO d_6_): δ 9.66 (dd, J = 5.3, 1.3 Hz, 2H, phen), 9.15 (dd, J = 8.2, 1.3 Hz, 2H, phen), 8.45 (s, 2H, phen), 8.33 (dd, J = 8.2, 5.3 Hz, 2H, phen).

IR spectroscopy: ν(NO): 1895, 1882 (KBr).

#### 3.1.4. Synthesis of fac-[RuNO(bpy)Cl_3_] (**4**)

The K_2_[RuNOCl_5_] 386 mg (1 mmol) sample and the ligand sample (1 mmol of bpy, 156 mg) were dissolved in a mixture of 45 mL EtOH and 15 mL H_2_O. The mixture was heated under a watch glass for 1 h. Further evaporation to 5 mL resulted in an orange precipitate of fac-[RuNO(bpy)Cl_3_] (**4**), which was filtered and dried in air. Yield was 69%.

Elemental analysis data (%) calc: C—30.5, H—2.1, N—10.7; found: C—30.0, H—2.0, N—10.3.

^1^H NMR (500 MHz, DMSO d_6_): δ 9.39 (d, J = 5.6 Hz, 2H, bpy), 8.88 (d, J = 8.0 Hz, 2H, bpym), 8.51 (td, J = 7.8, 1.2 Hz, 2H, bpy), 7.99 (ddd, J = 7.1, 5.8, 1.0 Hz, 2H, bpy).

IR spectroscopy: ν(NO): 1890, 1877 (KBr).

Spectral data for the compound **4** corresponds to that provided in [17].

#### 3.1.5. Synthesis of mer-[RuNO(bpy)Cl_3_] (**5**)

The K_2_[RuNOCl_5_] 386 mg (1 mmol) sample and the ligand sample (2 mmol of bpy, 312 mg) were dissolved in 40 mL H_2_O. The mixture was heated under a watch glass for 1 h. Further evaporation to 5 mL resulted in the green precipitate of mer-[RuNO(bpy)Cl_3_] (**5**), which was filtered and dried in air. Yield was 78%.

Elemental analysis data (%) calc: C—30.5, H—2.1, N—10.7; found: C—30.6, H—2.0, N—10.7.

^1^H NMR (500 MHz, DMSO d_6_): δ 9.52 (dd, J = 5.8, 1.3 Hz, 1H, bpy), 9.04 (dd, J = 5.8, 1.3 Hz, 1H, bpy), 8.86 (t, J = 7.3 Hz, 2H, bpy), 8.51 (td, J = 7.8, 1.5 Hz, 1H, bpy), 8.41 (td, J = 7.8, 1.5 Hz, 1H, bpy), 8.00 (ddd, J = 7.3, 5.8, 1.3 Hz, 1H, bpy), 7.86 (ddd, J = 7.3, 5.8, 1.3 Hz, 1H, bpy).

IR spectroscopy: ν(NO): 1866 (KBr).

Spectral data for the compound **5** corresponds to that provided in [17].

### 3.2. Physical Methods

Elemental analysis (C, H and N) was performed on a CE-440 analyzer (Exeter Analytical, Inc., North Chelmsford, MA, USA). IR spectra of the samples were measured in KBr pellets on an FSM 2201 IR Fourier spectrometer (OKB-SPECTR LLC, Saint-Petersburg, Russia) within the wavenumber range 400–4000 cm^−1^.

#### 3.2.1. Single-Crystal X-Ray Diffraction

Single-crystal X-ray diffraction data of **1**–**3**, **6** were collected on a Bruker Nonius X8Apex CCD area-detector, Bruker Kappa Apex II CCD area-detector and Bruker D8 Venture diffractometer with a CMOS PHOTON III detector (Bruker AXS GmbH, Karlsruhe, Germany) using graphite-monochromated MoKα radiation (λ = 0.71073 Å) via 0.5° ω- and ϕ-scan techniques. Experimental data reduction was performed using the APEX2 (Version 2008. 6-1) suite. The structures were solved by SHELXT-2019 and refined by the full-matrix least-squares technique SHELXL-2019, assisted by Olex2 GUI (Version 1.5). Atomic displacement parameters of the non-H atoms were refined in anisotropic approximation. Hydrogen atoms of organic ligands were located geometrically and refined using the riding model. All structures were deposited using the CCDC, with deposition numbers 2562801, 2562117, 2562118, 2566168.

#### 3.2.2. Nuclear Magnetic Resonance Spectroscopy

NMR spectra were recorded on a Bruker Avance III 500 spectrometer (Bruker AXS GmbH, Karlsruhe, Germany) operating at 500.03 MHz for ^1^H. Spectra were recorded in DMSO-d6 solution. The residual protons of the DMSO methyl group (δ = 2.5 ppm) were used as the internal standard. Spectra of **1**–**5** were recorded for samples containing 0.6 mL of a 10^−2^ M solution of the complexes in DMSO d_6_.

#### 3.2.3. Density Functional Theory Calculations

Density functional theory calculations of the **1**–**5** complex cations were performed using the ORCA 6.0.0 package. For the geometry optimization of complexes (closed-shell electron configuration), the B3LYP functional with the ZORA-def2-TZVP basis set was used. The SARC-ZORA-TZVP basis set was used for the ruthenium atom. Quantum chemical calculations of the isomeric forms for all complexes were performed with the CPCM solvation model (DMSO).

#### 3.2.4. UV-Vis and IR-Spectroscopy

UV-Vis spectra were recorded on an SF2000 UV-Vis single-beam spectrophotometer (OKB-SPECTR LLC, Saint-Petersburg, Russia). UV-Vis spectra of the complex were measured in a 1 cm quartz flow cell in dimethyl sulfoxide (DMSO). The absorbance of the pure solvents was considered. The concentration of the complex used for the photolysis measurements was 5 ×·10^−3^ M. Evolution of the UV-Vis spectra was monitored during exposure of the cuvette by a flow-through system (5 mL volume) using LED 450 nm, 100 mW. An optical power meter THORLABS PM16-401 (Thorlabs, Inc., Newton, NJ, USA) was used for the measurement of the light intensity. The detailed technique used in the united treatment of IR and UV-Vis data was described earlier [21].

#### 3.2.5. Electrospray Ionization–Mass Spectrometry

Mass spectrometric data were obtained on an Agilent liquid chromatograph–mass spectrometer system (LC-MS) 6130 Quadrupole MS (Agilent, Santa Clara, CA, USA), 1260 infinity LC. The analyses were performed in an *m*/*z* range from 100 to 1500 mass units, in SCAN mode, for positive and negative ions. Electrospray ionization (ESI) was used as an ion source. The following parameters were used: nitrogen as a drying gas, a temperature of 350 °C, a flow rate of 7 L min-1, a spraying gas (nitrogen) pressure of 60 psig, and a voltage on the capillary of 4000 V. The voltage on the fragmentator was established to be 100 V in all experiments. A 5 µL DMSO:EtOH 1:1 solution of the studied compound with a concentration of 2.5 × 10^−3^ M was injected into the mobile phase at a rate of 0.4 mL min^−1^, sprayed, and ionized. The mass spectra (MS) were interpreted by matching signals to the proposed ions, including a comparison of calculated and experimental isotopic peak distributions.

## 4. Conclusions

We used a simple technique for the preparation fac/mer-isomers of [RuNO(L)Cl_3_] (L = bpym, bpy, phen), starting from K_2_[RuNOCl_5_] as a suitable precursor. Depending on the ligand and preparation details, different isomers can be separated (NMR pure). In case of bipyrimidine, only the fac-isomer can be prepared with yields higher than 80%. For phenanthroline, the isomers can be separated depending on their solubility—the dominating mer-isomer precipitates first, and further evaporation of the main liquid can result in minor amounts of fac-isomer. For the bipyridine the ratio Ru:L and the solvent’s nature can drive the direction of the substitution. All complexes are stable in DMSO solutions and do not undergo isomer conversion, in contrast to [RuNO(NH_3_)_2_L(NO_3_)]^2+^. The photochemical NO release induced by 450 nm irradiation results in the substitution of NO in solvent ligands. The highest quantum yield (5.5%) was observed for the fac-[RuNO(bpym)Cl_3_] and the general trend shows that fac-isomers of all compounds are more sensitive to irradiation. The crystal structure of the photolysis product mer-[Ru(CH_3_CN)(bpy)Cl_3_] was determined by SC-XRD and the comparison with initial mer-[RuNO(bpy)Cl_3_] shows that the photoinduced NO release also does not change the conformation of the ruthenium coordination sphere.

## Figures and Tables

**Table 1:** Selected structural parameters: interatomic distances (Å) and angles (°) for structures **1**–**3** measured at 150 K.

	1 (*fac*-bpym)	2 (*fac*-phen)	3 (*mer*-phen)
Space group	*P*2_1_/n	*P*2_1_2_1_2_1_	*P*2_1_/n
d(N-O), Å	1.132 (2)	1.139 (4)	1.132 (5)
∠(Ru-N-O), °	168.90 (16)	170.8 (3)	172.4 (4)
d(Ru-NO), Å	1.7435 (17)	1.746 (3)	1.742 (4)
d(Ru-Cl), Å	2.3572 (5) 2.3628 (5) 2.3462 (5) (trans)	2.3759 (10) 2.3813 (9) 2.3460 (10) (trans)	2.3624 (14) 2.3502 (15) 2.3586 (14)
d(Ru-N(L)), Å	2.0815 (16) 2.0816 (16)	2.076 (3) 2.082 (3)	2.084 (4) 2.097 (5) (trans)

**Table 2:** Photodissociation quantum yields for compounds **1**–**5** measured in DMSO (solvent variations are noted where applicable).

Compound	QY, %
*fac*-[RuNO(bpym)Cl_3_] (**1**)	5.4 ± 0.5
*fac*-[RuNO(phen)Cl_3_] (**2**)	0.8 ± 0.1
*mer*-[RuNO(phen)Cl_3_] (**3**)	0.6 ± 0.1
*fac*-[RuNO(bpy)Cl_3_] (**4**)	3.7 ± 0.1
*mer*-[RuNO(bpy)Cl_3_] (**5**)	2.1 ± 0.1/2.4 ± 0.1 (DMSO/CH_3_CN)

**Table 3:** Correlation between the relative transition contribution and the quantum yield for compounds **2**–**5**.

Complex	RC, %	QY, %
**2** (*fac*-)	32	0.8 ± 0.1
**3** (*mer*-)	23	0.6 ± 0.1
**4** (*fac*-)	63	3.7 ± 0.1
**5** (*mer*-)	44	2.1 ± 0.1

**Table 4:** Proposed assignment of predominant fragment ions in the ESI mass spectra of compounds **1**–**5** in 1:1 DMSO:EtOH solutions.

Compound	*m*/*z*	Proposed Fragment Composition
*fac*-[RuNO(bpym)Cl_3_] (**1**)	529	[Ru(bpym)(DMSO)_3_Cl]^+^
*fac*-[RuNO(phen)Cl_3_] (**2**)	510	[Ru(phen)(DMSO)_2_Cl_2_]^+^
*mer*-[RuNO(phen)Cl_3_] (**3**)		
*fac*-[RuNO(bpy)Cl_3_] (**4**)	486	[Ru(bpy)(DMSO)_2_Cl_2_]^+^
*mer*-[RuNO(bpy)Cl_3_] (**5**)		

**Table 5:** Selected structural parameters: interatomic distances (Å) and angles (°) for structure **6** measured at 100 K.

	6	5 *
Space group	P-1	Pn
∠(Ru-N-O(C)), °	173.3 (7)	174.01
d(Ru-N), Å	2.056 (7)	1.938
d(Ru-Cl), Å	2.353 (2) 2.363 (2) 2.314 (2)	2.356 2.349 2.336
d(Ru-N(L)), Å	2.054 (7) 2.037 (6) (trans)	2.079 2.100 (trans)

* The data were taken from CCDC code ZACLUK.

## Data Availability

Data is contained within the article or Supplementary Material.

## Supplementary Materials

The following supporting information can be downloaded at https://www.mdpi.com/article/10.3390/ijms27146172/s1.
