# Supplementary material for: Ruthenium Nitrosyl Complexes with Bidentate Heterocycles and Chloride Ligands: Synthesis and Photorelease of NO
Source: Int J Mol Sci. 2026 Jul 10;27(14):6172. doi: 10.3390/ijms27146172 (PMC13409931; doi:10.3390/ijms27146172)
Supplement: Supplementary file 1 [file ijms-27-06172-s001.zip › ijms-4441511-supplementary.pdf]

Supporting information

Ruthenium Nitrosyl Complexes with Bidentate Heterocycles and Chloride Ligands: Synthesis and Photorelease of NO

Anastasia O. Brovko [1], Ivan A. Yakovlev [1], Natalia V. Kuratieva [1,2], Dmitriy G. Sheven [1] and Gennadiy A. Kostin [1,*]

[1] Nikolaev Institute of Inorganic Chemistry, Siberian Branch of the Russian Academy of Sciences, 3 Acad. Lavrentiev Avenue, 630090 Novosibirsk, Russia

[2] N.N. Vorozhtsov Novosibirsk Institute of Organic Chemistry, Siberian Branch of the Russian Academy of Sciences, 9 Acad. Lavrentiev Avenue, 630090 Novosibirsk, Russia

*Corresponding author: kostin@niic.nsc.ru

The structural information of complexes 1-3

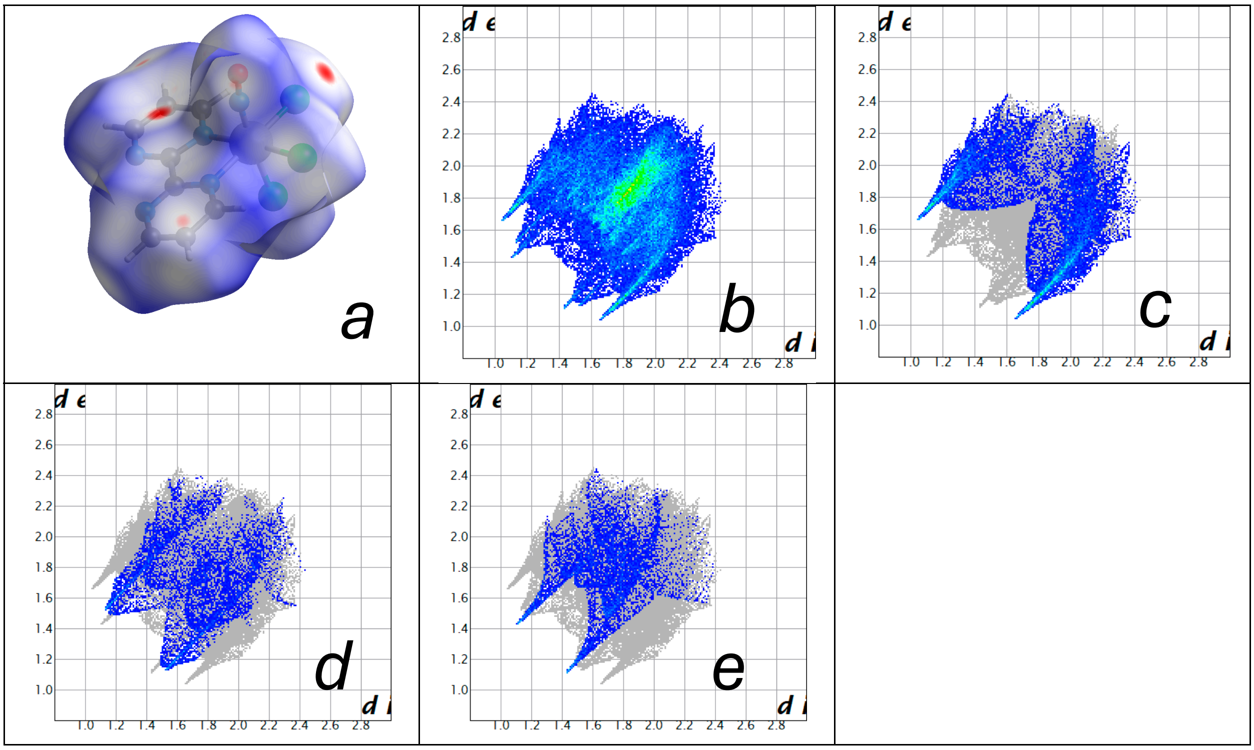

Figure S1. Hirshfield surface (a) and fingerprints (b – interactions of all atoms, c – Cl-H interactions (23.9 %), d – N-H interactions (11.0 %), e – O-H interactions (11.0 %)) of *fac*-[RuNO(*bpym*)Cl$_3$] **(*1*)**.

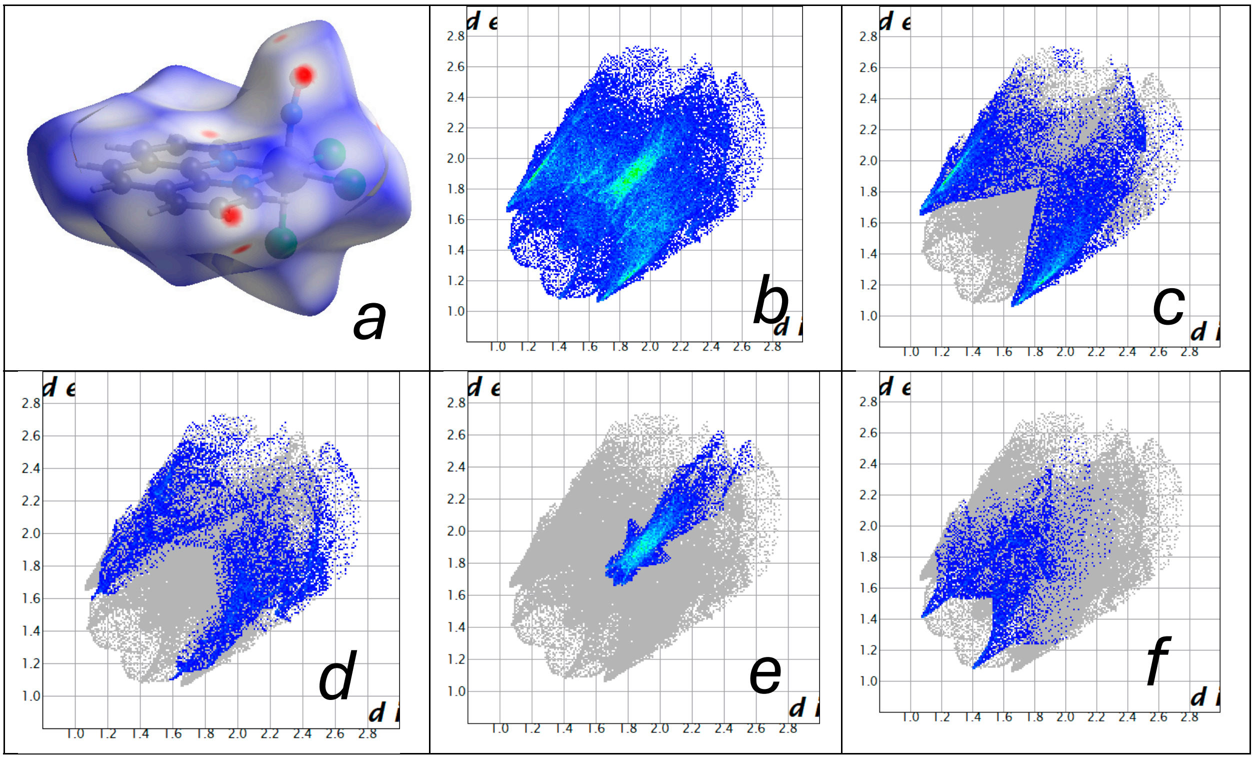

Figure S2. Hirshfield surface (a) and fingerprints (b – interactions of all atoms, c – Cl-H interactions (27,1 %), d – C-H interactions (16,6 %), e – Cl-C interactions (13,4 %), f – O-H interactions (9,4)) of fac-[RuNO(phen)Cl_3] **(2)**.

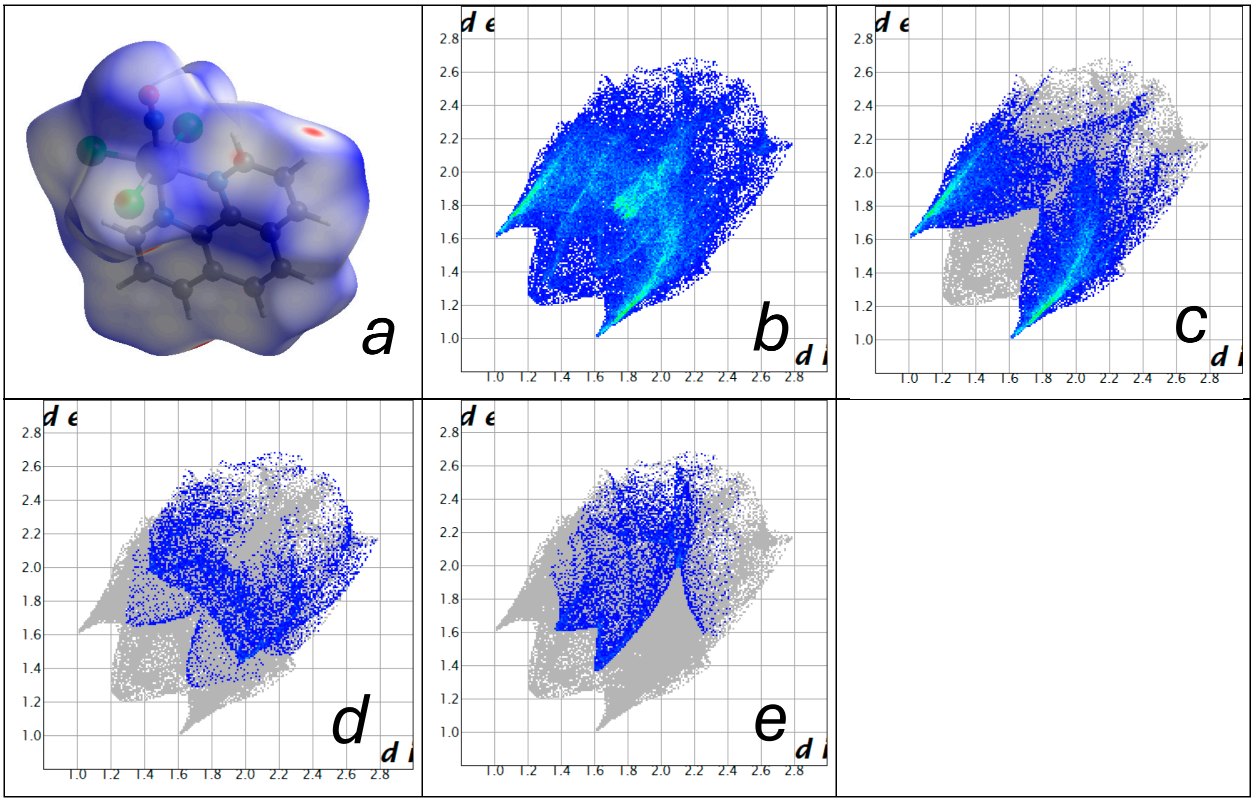

Figure S3. Hirshfield surface (a) and fingerprints (b – interactions of all atoms, c – Cl-H interactions (42,0 %), d – C-H interactions (10,4 %), e – O-H interactions (9,2 %)) of mer-[RuNO(phen)Cl_3] **(3)**.

Solution Studies

1H NMR spectra of fac-[RuNO(bpym)Cl$_3$] (1)

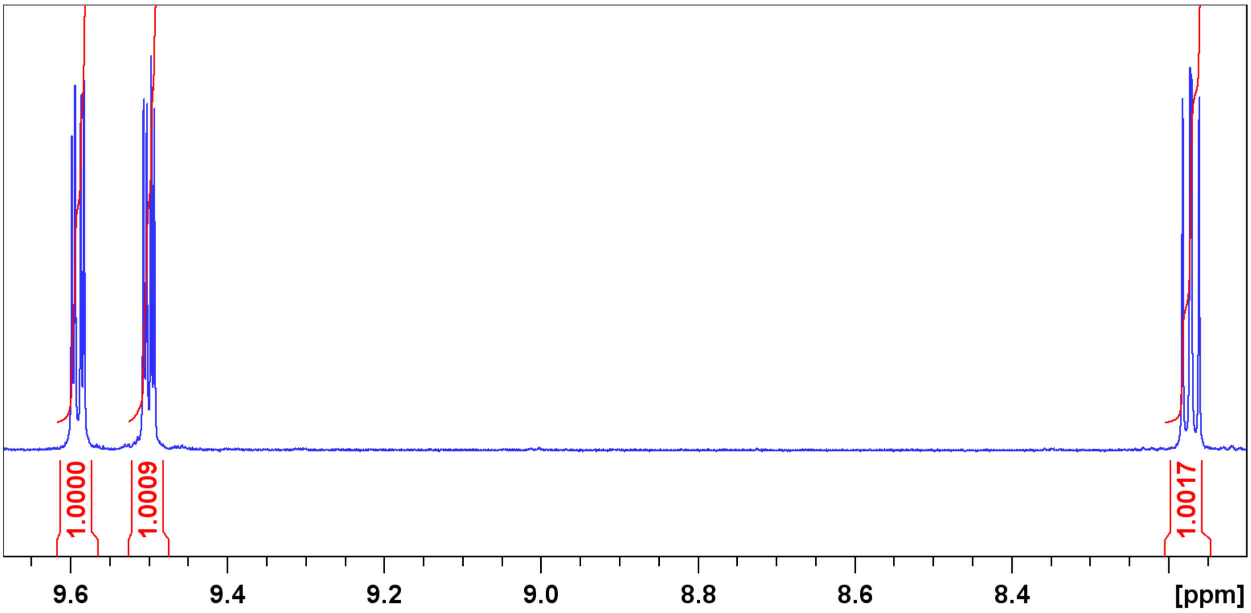

Figure S4. *^{1}H NMR spectra of DMSO solution of **1**.*

1H NMR (500 MHz, DMSO-d$_6$): δ 9.59 (dd, J=5.7, 2.1 Hz, 2H, bpym), 9.50 (dd, J=4.8, 2.1 Hz, 2H, bpym), 8.17 (dd, J=5.7, 4.8 Hz, 2H, bpym), 3.31 (br s, H_2O, residual water in the solvent), 2.50 (quintet, J=2.5 Hz, DMSO).

Full-range 1H NMR spectra of 1

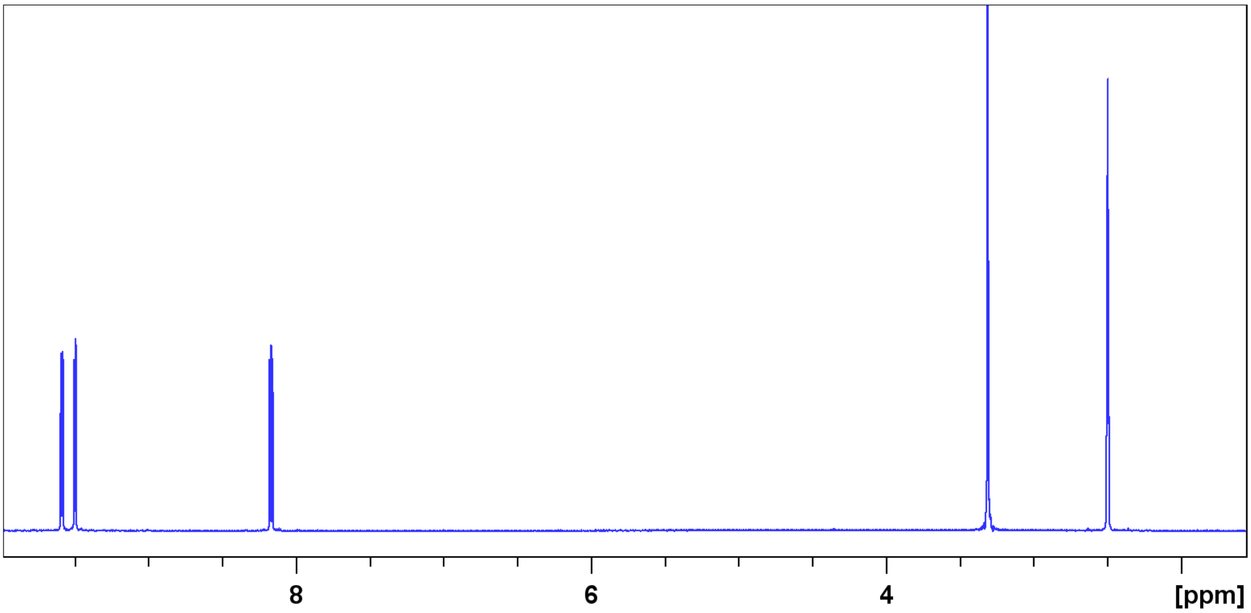

1H NMR spectra of fac-[RuNO(phen)Cl₃] (2)

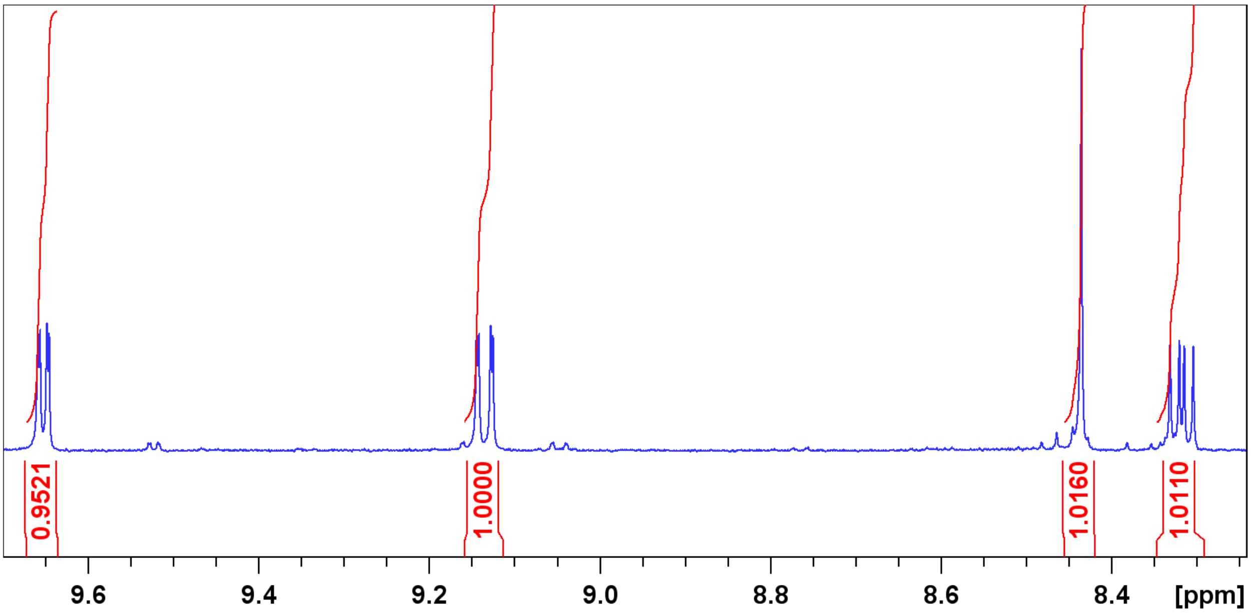

Figure S5. *^{1}H NMR spectra of DMSO solution of* ***2****.*

1H NMR (500 MHz, DMSO-d_6): δ 9.66 (dd, J=5.3, 1.3 Hz, 2H, phen), 9.15 (dd, J=8.2, 1.3 Hz, 2H, phen), 8.45 (s, 2H, phen), 8.33 (dd, J=8.2, 5.3 Hz, 2H, phen), 3.32 (br s, H_2O, residual water in the solvent), 2.50 (quintet, J=2.5 Hz, DMSO).

Full-range 1H NMR spectra of 2

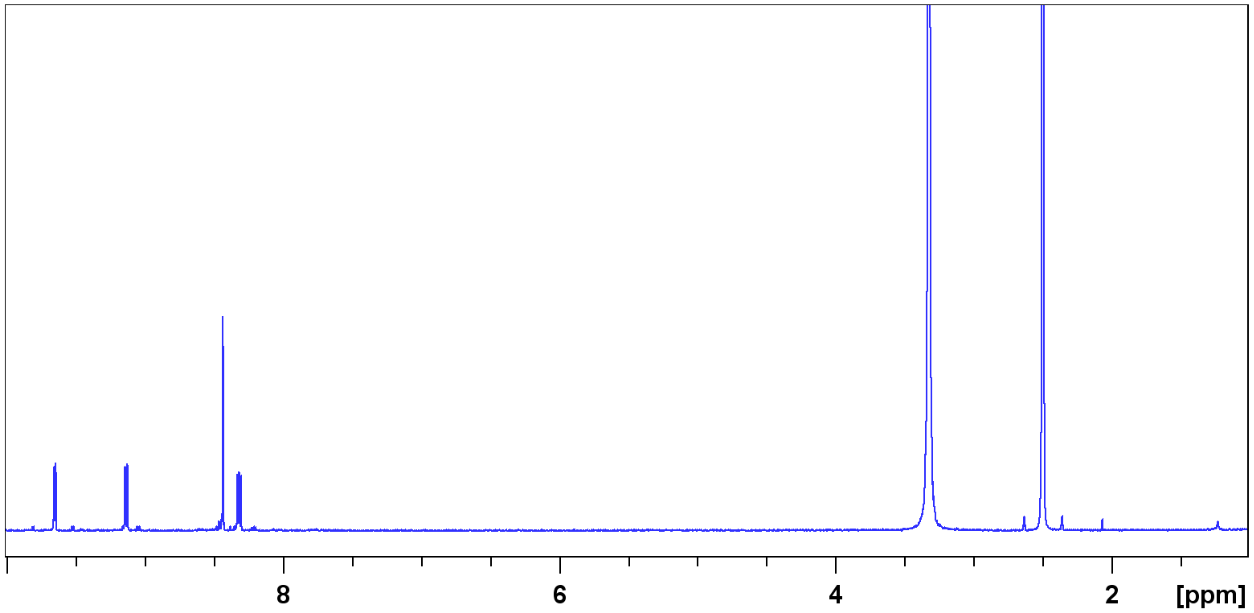

1H NMR spectra of mer-[RuNO(phen)Cl₃] (3)

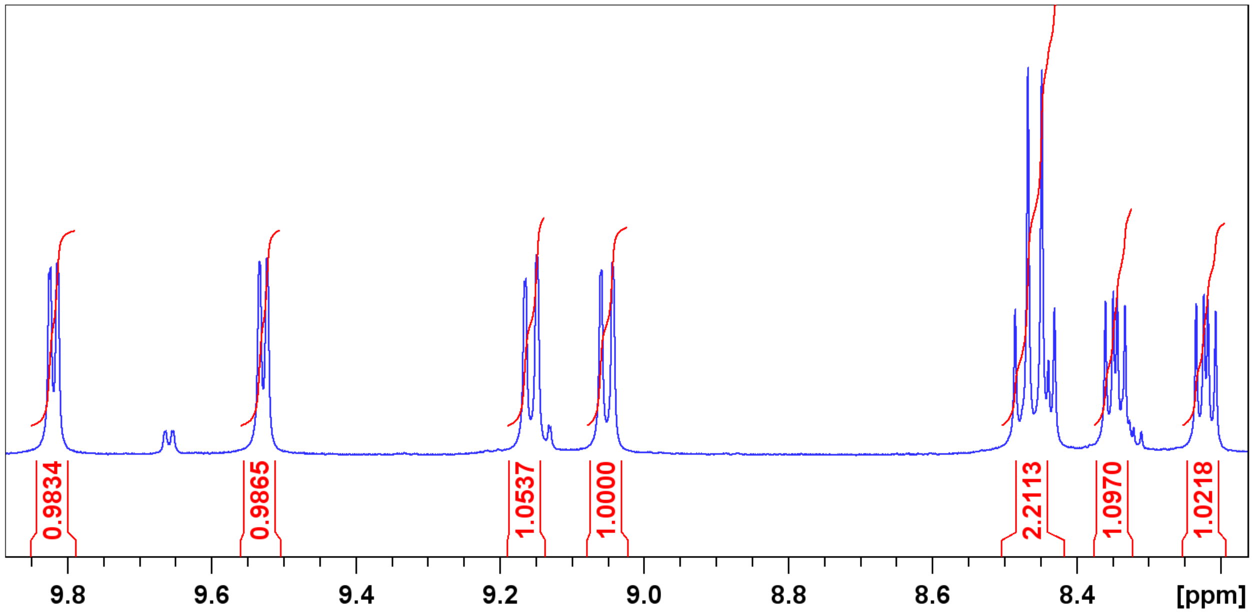

Figure S6. *^{1}H NMR spectra of DMSO solution of* ***3****.*

1H NMR (500 MHz, DMSO-d_6): δ 9.82 (dd, J=5.3, 0.9 Hz, 1H, phen), 9.53 (d, J=5.3 Hz, 1H, phen), 9.16 (dd, J=8.2, 1.0 Hz, 1H, phen), 9.06 (dd, J=8.2, 0.9 Hz, 1H, phen), 8.47 (t, J=8.9 Hz, 1H, phen), 8.45 (t, J=8.9 Hz, 1H, phen), 8.35 (dd, J=8.2, 5.3 Hz, 1H, phen), 8.22 (dd, J=8.2, 5.3 Hz, 1H, phen), 3.32 (br s, H_2O, residual water in the solvent), 2.50 (quintet, J=2.5 Hz, DMSO).

Full-range 1H NMR spectra of 3

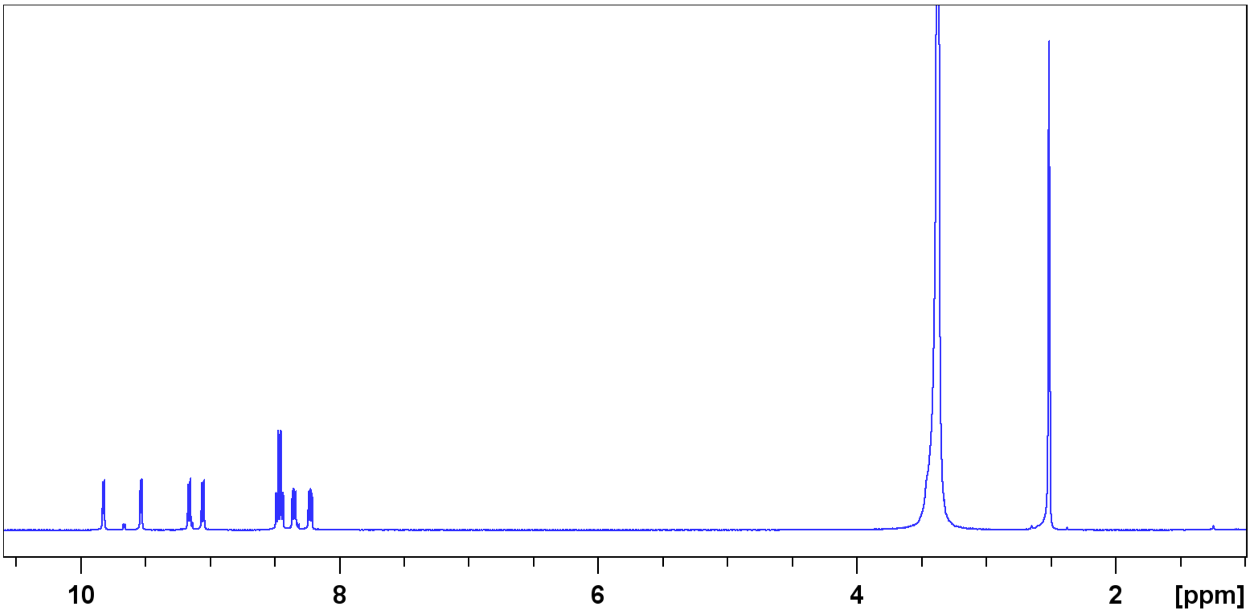

1H NMR spectra of fac-[RuNO(bpy)Cl_3] (4)

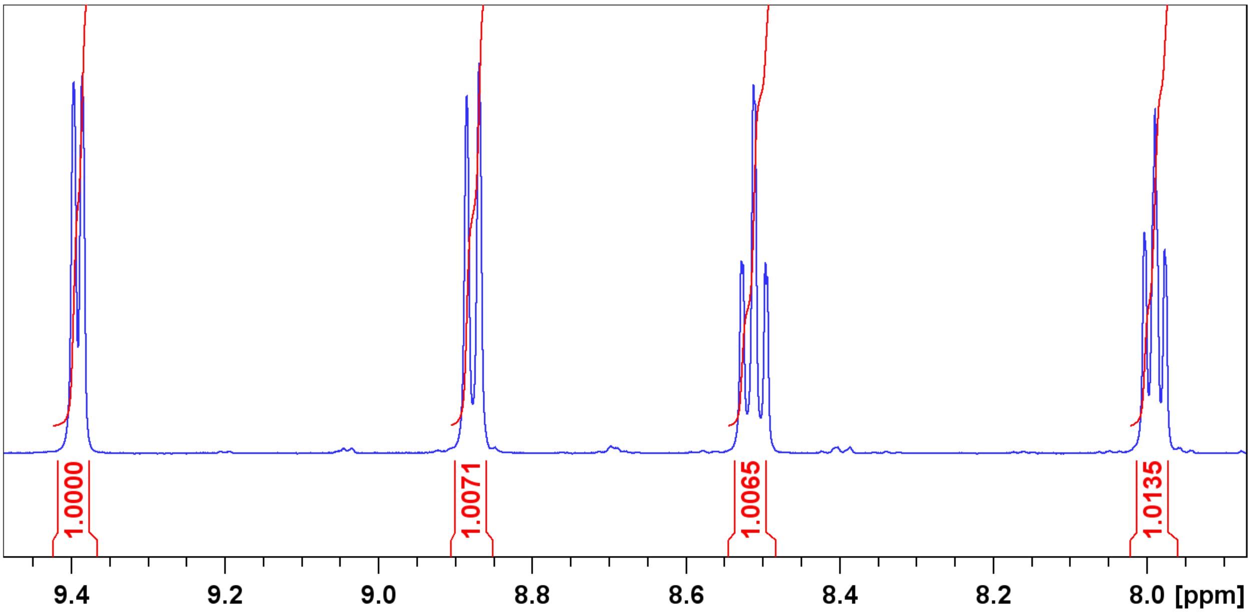

Figure S7. *^{1}H NMR spectra of DMSO solution of **4**.*

1H NMR (500 MHz, DMSO-d_6): δ 9.39 (d, J=5.6 Hz, 2H, bpy), 8.88 (d, J=8.0 Hz, 2H, bpym), 8.51 (td, J=7.8, 1.2 Hz, 2H, bpy), 7.99 (ddd, J=7.1, 5.8, 1.0 Hz, 2H, bpy), 3.32 (br s, H_2O, residual water in the solvent), 2.50 (quintet, J=2.5 Hz, DMSO).

Full-range 1H NMR spectra of 4

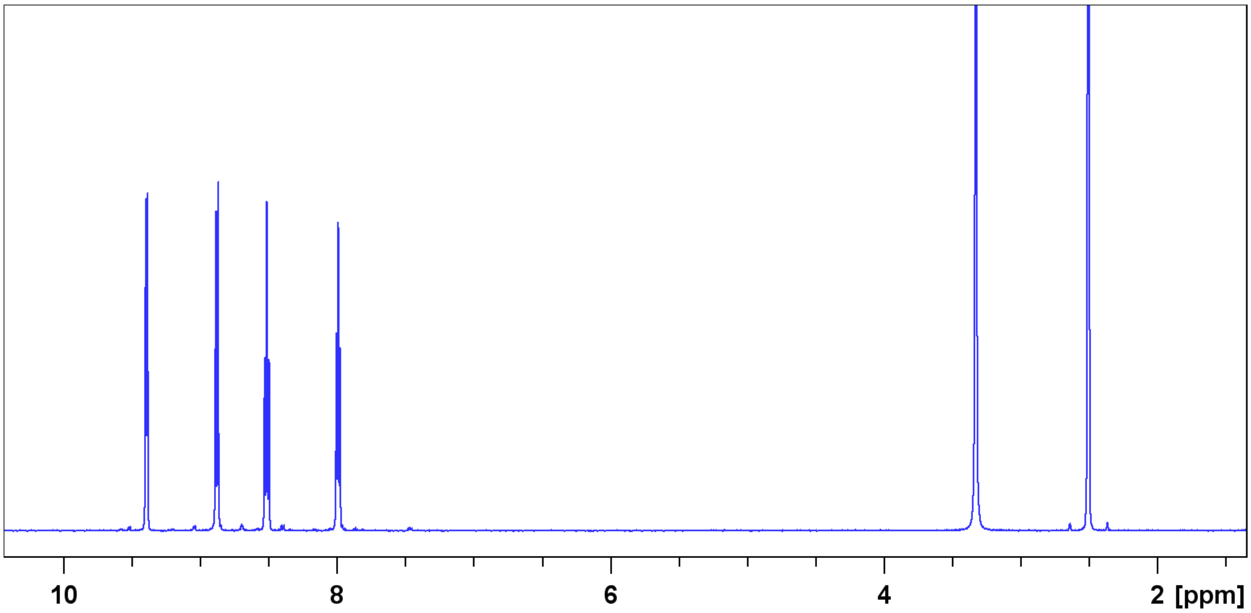

1H NMR spectra of mer-[RuNO(bpy)Cl$_3$] (5)

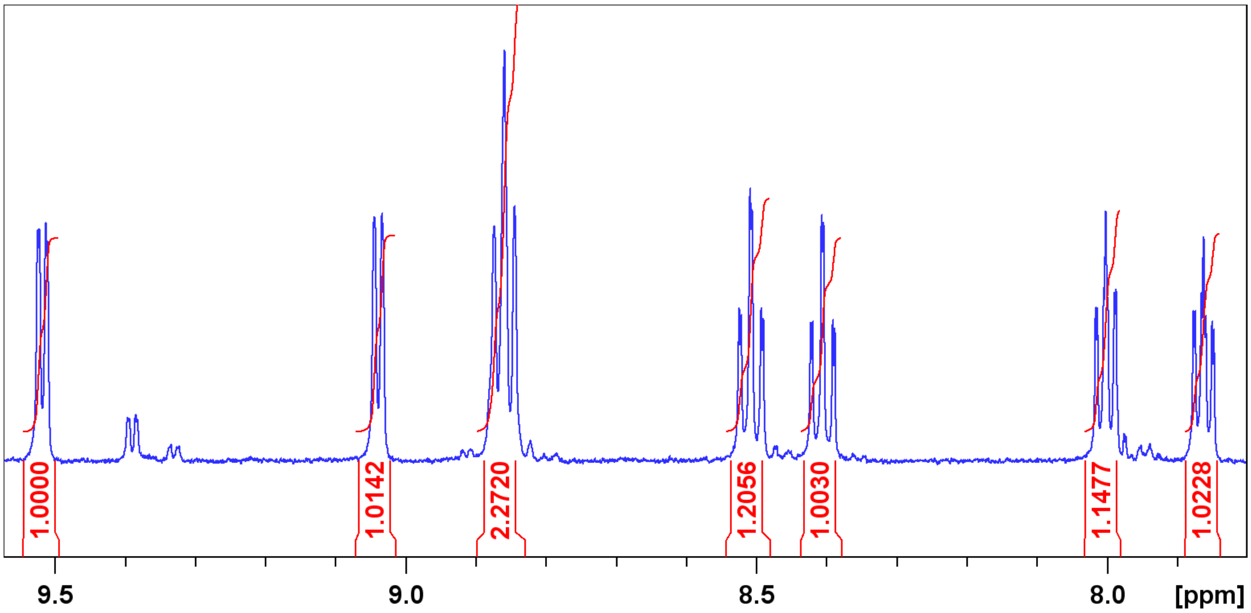

Figure S8. *^{1}H NMR spectra of DMSO solution of* ***5***.

1H NMR (500 MHz, DMSO-d$_6$): δ 9.52 (dd, J=5.8, 1.3 Hz, 1H, bpy), 9.04 (dd, J=5.8, 1.3 Hz, 1H, bpy), 8.86 (t, J=7.3 Hz, 2H, bpy), 8.51 (td, J=7.8, 1.5 Hz, 1H, bpy), 8.41 (td, J=7.8, 1.5 Hz, 1H, bpy), 8.00 (ddd, J=7.3, 5.8, 1.3 Hz, 1H, bpy), 7.86 (ddd, J=7.3, 5.8, 1.6 Hz, 1H, bpy), 3.33 (br s, H_2O, residual water in the solvent), 2.50 (quintet, J=2.5 Hz, DMSO).

Full-range 1H NMR spectra of 5

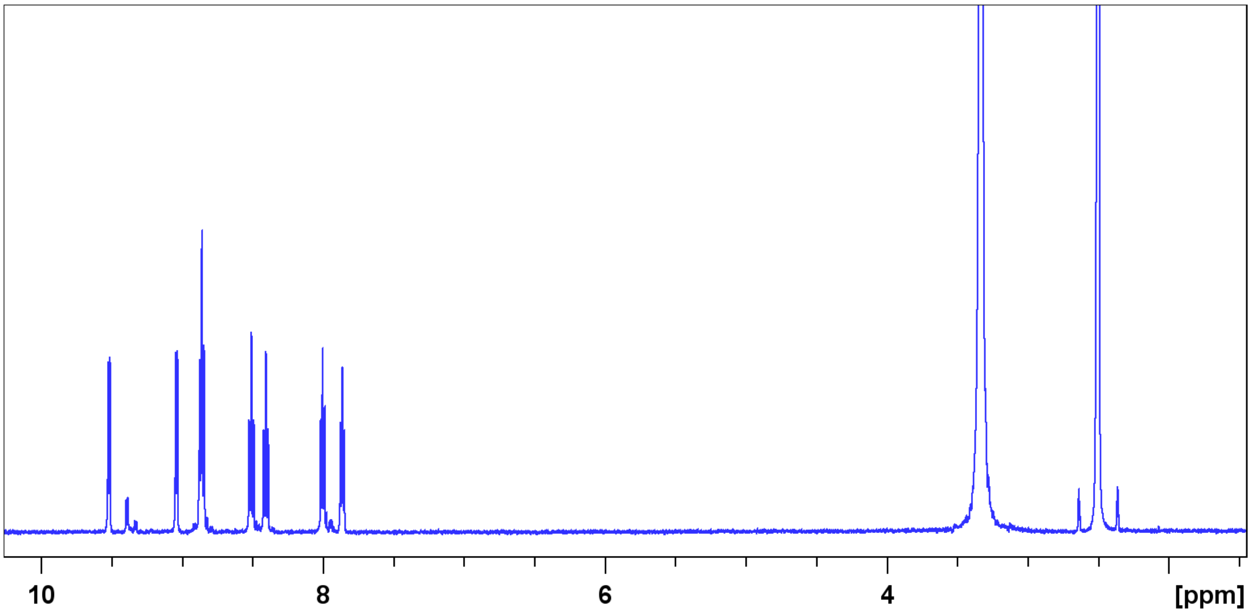

Photoinduced NO release

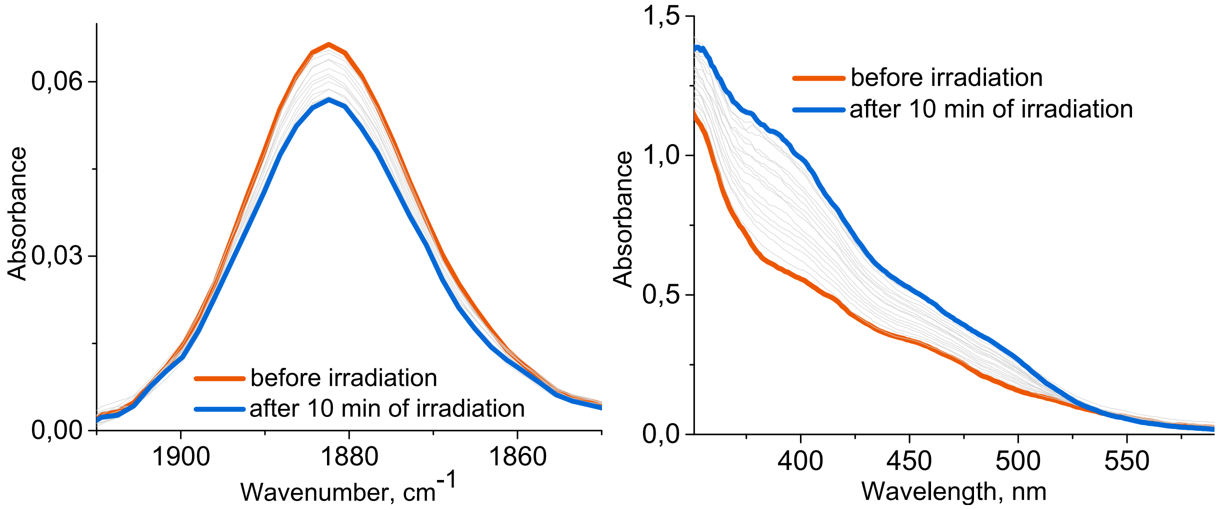

Figure S9. *Evolution of IR (left) and UV-Vis (right) spectra for DMSO solution of compound fac-[RuNO(phen)Cl$_3$] **(2)** under continuous 450 nm irradiation for 10 minutes.*

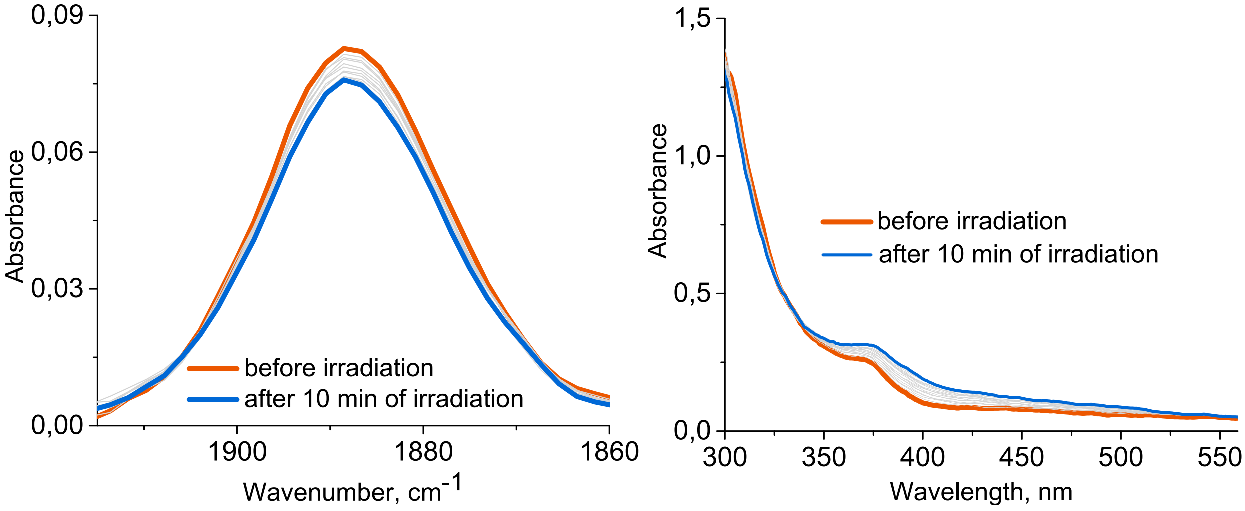

Figure S10. *Evolution of IR (left) and UV-Vis (right) spectra for DMSO solution of compound mer-[RuNO(phen)Cl₃]* ***(3)*** *under continuous 450 nm irradiation for 10 minutes.*

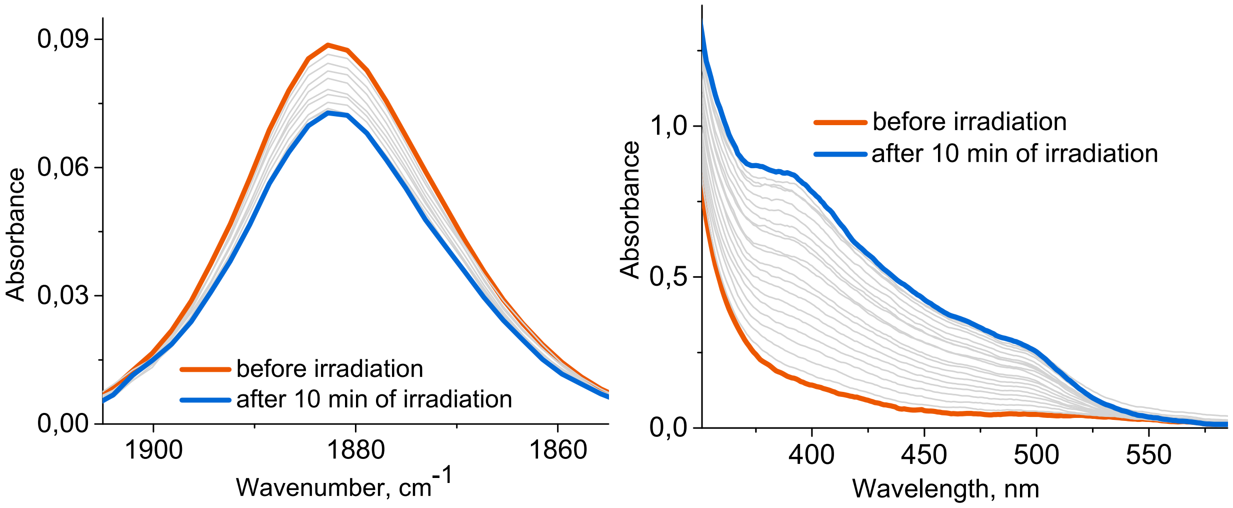

Figure S11. *Evolution of IR (left) and UV-Vis (right) spectra for DMSO solution of compound fac-[RuNO(bpy)Cl₃]* ***(4)*** *under continuous 450 nm irradiation for 10 minutes.*

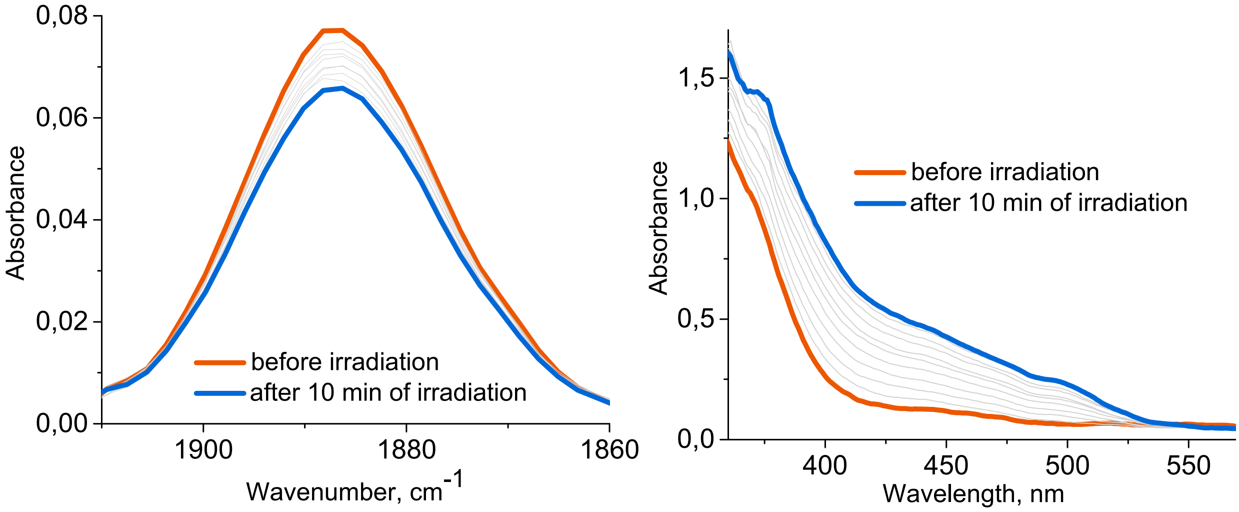

Figure S12. *Evolution of IR (left) and UV-Vis (right) spectra for DMSO solution of compound mer-[RuNO(bpy)Cl₃]* ***(5)*** *under continuous 450 nm irradiation for 10 minutes.*

TD-DFT calculations

Table S1. *The transitions closest to the irradiation wavelength and their orbital contributions fac-[RuNO(bpym)Cl₃] (**1**)*

λ	Orbital contributions (>10%)
441	HOMO-5 → LUMO : 0.56<br>HOMO-4 → LUMO : 0.09<br>HOMO-2 → LUMO : 0.24
436.9	HOMO-4 → LUMO : 0.30<br>HOMO-3 → LUMO : 0.41<br>HOMO-3 → LUMO+1 : 0.08
430.2	HOMO-2 → LUMO+2 : 0.09<br>HOMO-1 → LUMO : 0.32<br>HOMO-1 → LUMO+1 : 0.31
426.2	HOMO-4 → LUMO : 0.21<br>HOMO-2 → LUMO : 0.32<br>HOMO-2 → LUMO+1 : 0.12<br>HOMO-1 → LUMO+2 : 0.17
423.7	HOMO → LUMO+3 : 0.85

Table S2. *The transitions closest to the irradiation wavelength and their orbital contributions for fac-[RuNO(phen)Cl₃] (**2**) and mer-[RuNO(phen)Cl₃] (**3**)*

fac-[RuNO(phen)Cl_3] (**2**)		*mer*-[RuNO(phen)Cl_3] (**3**)	
λ	Orbital contributions (>10%)	λ	Orbital contributions (>10%)
520.3	HOMO-6 → LUMO : 0.11<br>HOMO-5 → LUMO : 0.13<br>HOMO-4 → LUMO : 0.10<br>HOMO-2 → LUMO+1 : 0.15<br>HOMO-1 → LUMO : 0.30	457	HOMO-3 -> LUMO+1 : 0.38<br>HOMO-2 -> LUMO+1 : 0.49
515.3	HOMO-6 → LUMO: 0.11<br>HOMO-5 → LUMO : 0.39<br>HOMO-4 → LUMO : 0.16<br>HOMO-1 → LUMO : 0.14	433.2	HOMO -> LUMO+3 : 0.84
424.9	HOMO-3 → LUMO+1 : 0.85	411.7	HOMO-6 -> LUMO : 0.90
408.6	HOMO-6 → LUMO : 0.24<br>HOMO-5 → LUMO+1 : 0.29<br>HOMO-4 → LUMO : 0.15<br>HOMO-4 → LUMO+1 : 0.12	408	HOMO-5 -> LUMO : 0.16<br>HOMO-4 -> LUMO+1 : 0.38<br>HOMO-1 -> LUMO : 0.15
395.3	HOMO-5 → LUMO : 0.16<br>HOMO-4 → LUMO : 0.41<br>HOMO-4 → LUMO+1 : 0.09<br>HOMO-3 → LUMO+1 : 0.01	407.5	HOMO-6 -> LUMO+1 : 0.48<br>HOMO-5 -> LUMO : 0.24

Table S3. *The transitions closest to the irradiation wavelength and their orbital contributions for fac-[RuNO(bpy)Cl₃]* ***(4)*** *and* mer-*[RuNO(bpy)Cl₃]* ***(5)***

fac-[RuNO(bpy)Cl_3] **(4)**		*mer*-[RuNO(bpy)Cl_3] **(5)**	
λ	Orbital contributions (>10%)	λ	Orbital contributions (>10%)
511.5	HOMO-6 → LUMO : 0.13<br>HOMO-5 → LUMO : 0.24<br>HOMO-3 → LUMO+1 : 0.19<br>HOMO-1 → LUMO : 0.26	488.4	HOMO-1 -> LUMO+1 : 0.97
418	HOMO-6 → LUMO : 0.56<br>HOMO-5 → LUMO : 0.08	464.8	HOMO-3 -> LUMO : 0.98
415.3	HOMO-4 → LUMO+1 : 0.21<br>HOMO-3 → LUMO+1 : 0.27<br>HOMO-2 → LUMO : 0.13<br>HOMO-1 → LUMO+1 : 0.26	438	HOMO-1 -> LUMO+3 : 0.39<br>HOMO-1 -> LUMO+4 : 0.41
394.8	HOMO-3 → LUMO : 0.54<br>HOMO-4 → LUMO : 0.12<br>HOMO-2 → LUMO : 0.18	435.4	HOMO-1 -> LUMO+1 : 0.89
393.6	HOMO-6 → LUMO+1 : 0.44<br>HOMO-5 → LUMO : 0.15<br>HOMO-3 → LUMO+1 : 0.10<br>HOMO-1 → LUMO+1 : 0.21	428.4	HOMO-1 -> LUMO+5 : 0.68<br>HOMO -> LUMO+6 : 0.24

Photoinduced NO release

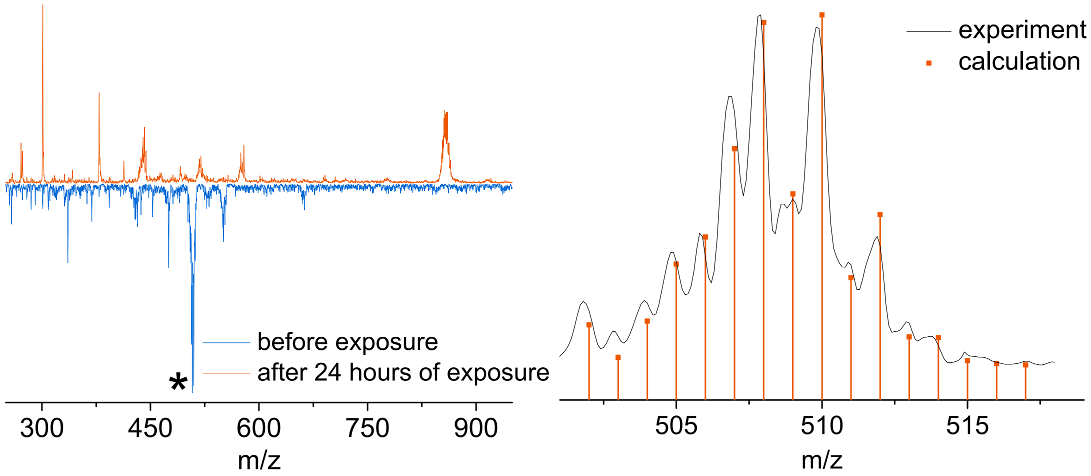

Figure S13. *ESI-MS spectra before and after light irradiation by 450 nm of mer-[RuNO(phen)Cl₃] (3) (left) and the peak 510 m/z from mass spectra of* ***3*** *with calculated isotope pattern (right)*

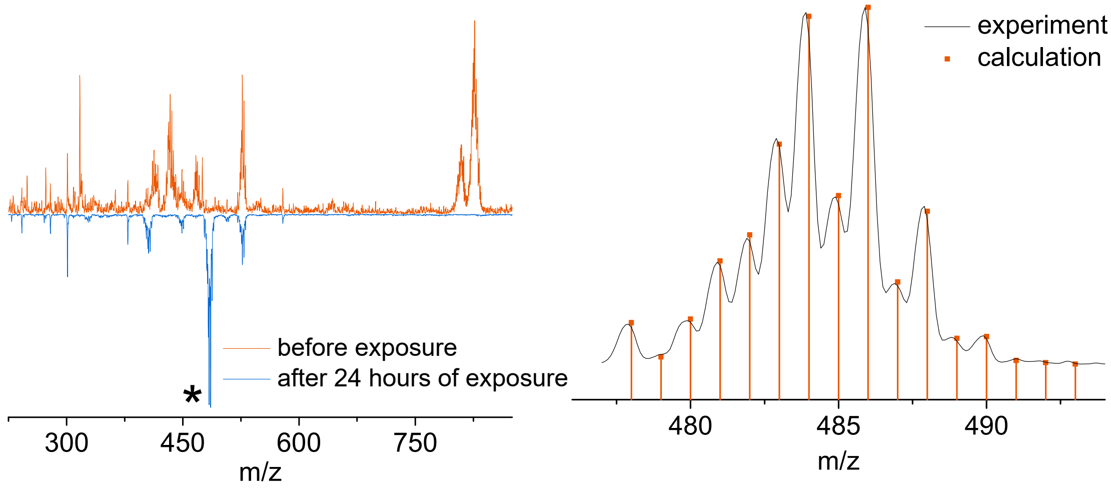

Figure S14. *ESI-MS spectra before and after light irradiation by 450 nm of fac-[RuNO(bpy)Cl*$_3$*] (**4**) (left) and the peak 486 m/z from mass spectra of **4** with calculated isotope pattern (right)*

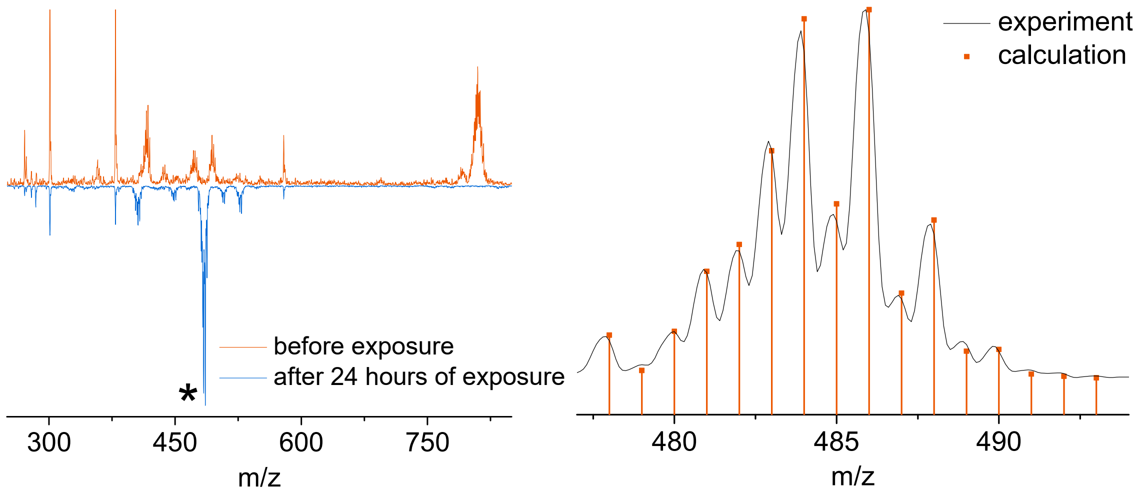

Figure S15. *ESI-MS spectra before and after light irradiation by 450 nm of mer-[RuNO(bpy)Cl*$_3$*] (**5**) (the left panel) and the peak 486 m/z from mass spectra of **5** with calculated isotope pattern (the right panel)*
